# The Proteomic Analysis of Platelet Extracellular Vesicles in Diabetic Patients by nanoLC-MALDI-MS/MS and nanoLC-TIMS-MS/MS

**DOI:** 10.3390/molecules30061384

**Published:** 2025-03-20

**Authors:** Joanna Kasprzyk-Pochopień, Agnieszka Kamińska, Przemysław Mielczarek, Radosław Porada, Ewa Stępień, Wojciech Piekoszewski

**Affiliations:** 1 Laboratory of High-Resolution Mass Spectrometry, Faculty of Chemistry, Jagiellonian University, 30-387 Krakow, Poland; joanna.m.kasprzyk@uj.edu.pl; 2 Department of Medical Physics, M. Smoluchowski Institute of Physics, Faculty of Physics, Astronomy and Applied Computer Science, Jagiellonian University, 30-348 Krakow, Poland; agnieszka.kaminska@ikifp.edu.pl (A.K.); e.stepien@uj.edu.pl (E.S.); 3 Department of Analytical Chemistry and Biochemistry, Faculty of Materials Science and Ceramics, AGH University of Krakow, 30-059 Krakow, Poland; przemyslaw.mielczarek@agh.edu.pl; 4 Department of Analytical Chemistry, Faculty of Chemistry, Jagiellonian University, 30-387 Krakow, Poland; radoslaw.porada@uj.edu.pl; 5 Total-Body Jagiellonian-PET Laboratory, Jagiellonian University, 30-348 Krakow, Poland; 6 Center for Theranostics, Jagiellonian University, 31-501 Krakow, Poland

**Keywords:** proteomic analysis, platelet extracellular vesicles (PEVs), diabetes mellitus, nanoLC-MALDI-MS/MS, nanoLC-TIMS-MS/MS, biomarker discovery

## Abstract

Platelet extracellular vesicles (PEVs) are emerging as key biomarkers in diabetes mellitus (DM), reflecting altered platelet function and coagulation pathways. This study compares two proteomic techniques—nanoLC-MALDI-MS/MS and nanoLC-TIMS-MS/MS—for analyzing PEVs in diabetic patients, to assess their potential for biomarker discovery. PEVs were isolated from platelet-rich plasma and characterized using tunable resistive pulse sensing (TRPS), Fourier-transform infrared (FTIR) spectroscopy, and transmission electron microscopy (TEM). Proteomic analyses identified significant differences in protein expression between diabetic and non-diabetic individuals, with nanoLC-TIMS-MS/MS demonstrating superior sensitivity by detecting 97% more unique proteins than nanoLC-MALDI-MS/MS. Key differentially expressed proteins included apolipoproteins and oxidative stress markers, which may contribute to platelet dysfunction and cardiovascular complications in DM. Sex-specific variations in protein expression were also observed, highlighting potential differences in disease progression between male and female patients. The integration of advanced proteomic methodologies provides novel insights into the role of PEVs in diabetes pathophysiology, underscoring their diagnostic and therapeutic potential. These findings pave the way for improved biomarker-based strategies for early detection and monitoring of diabetic complications.

## 1. Introduction

Diabetes mellitus (DM) is a metabolic disorder characterized by chronic hyperglycemia due to either autoimmune destruction of insulin-producing β-cells (Type 1 DM), insulin resistance (Type 2 DM), or glucose intolerance occurring during pregnancy (gestational DM) [1,2,3]. This disease results in widespread cellular imbalances affecting several organs, including the kidneys, liver, muscles, and adipose tissue [4]. Clinically, diabetes manifests through symptoms like polydipsia, polyuria, polyphagia, and unintended weight loss [2], while long-term complications include cardiovascular diseases, hypertension, vision impairment, nephropathy, and neuropathy [4,5]. Currently, more than 830 million people (in 2022) worldwide suffer from diabetes, making it the ninth leading cause of death globally [6,7]. The standard treatment approach includes the administration of insulin or hypoglycemic drugs aimed at controlling blood glucose levels [2]. However, these therapies are not always effective, as they primarily target individual organs, while diabetes affects the intricate signaling between multiple metabolic organs, such as the pancreas, liver, and brain [1].

Recent studies have highlighted the role of extracellular vesicles (EVs) as important mediators of intercellular communication in various physiological and pathological processes, including diabetes. EVs are nanosized, membrane-bound particles released by nearly all cell types, including platelets, endothelial cells, and immune cells [8,9,10]. Due to their diverse origins and molecular cargo, EVs have gained increasing attention as potential biomarkers and therapeutic targets in metabolic disorders.

EVs are classified into three major categories, based on their size and biogenesis: exosomes, ectosomes (also known as microvesicles), and apoptotic bodies [5,10]. Exosomes (40–100 nm) originate from the inward budding of the endosomal membrane, and are released upon fusion of multivesicular bodies with the plasma membrane [1,5]. Ectosomes (100–1000 nm), on the other hand, are formed by the outward budding of the plasma membrane, and this group includes platelet extracellular vesicles (PEVs). While some smaller PEVs may overlap in size with exosomes, their distinct biogenesis links them closely to the ectosome category. Lastly, apoptotic bodies (500–2000 nm) are larger vesicles that are shed during programmed cell death, and often contain nuclear material [10].

EVs are known to carry a variety of bioactive molecules, including proteins, nucleic acids (DNA, mRNA, and miRNA), lipids, and metabolites, reflecting the molecular profile of their cell of origin [1,11]. These molecular components enable EVs to modulate recipient cells, influencing key processes, such as inflammation, immune responses, and metabolic regulation, all of which are relevant in the context of diabetes pathophysiology [1,2,10,12].

In the context of diabetes, the unique proteomic profile of PEVs may provide valuable insights into disease progression and potential therapeutic targets. By carrying platelet-derived molecules linked to coagulation, inflammation, and vascular function, PEVs represent a promising avenue for discovering biomarkers associated with platelet dysfunction and diabetic complications.

Platelet extracellular vesicles (PEVs) are increasingly recognized as promising biomarkers in diabetes mellitus, due to their unique characteristics and strong association with disease pathology. PEVs are among the most abundant extracellular vesicles (EVs) in circulation, providing a stable and accessible target for analysis [13]. In diabetic patients, the production and composition of PEVs are significantly altered. Platelets—key players in blood coagulation and immune responses—release PEVs that serve as mediators of intercellular communication. Their number and molecular cargo are notably modulated in the diabetic state [1,7].

This alteration is reflected in an increased release of PEVs, indicating a state of chronic platelet activation, which contributes to the hypercoagulable state commonly observed in diabetes [14]. Moreover, EVs from activated platelets have been shown to exhibit high procoagulant activity, estimated to be 50–100 times higher than the procoagulant activity of intact platelets [15]. This heightened activity is closely linked to vascular dysfunction in diabetic patients.

Altered PEVs in diabetes often carry pro-inflammatory and pro-thrombotic factors that exacerbate diabetic complications, such as cardiovascular disease, nephropathy, and diabetic wounds [1,2]. Their involvement in coagulation, inflammation, and vascular dysfunction underscores their potential as valuable biomarkers for monitoring disease progression and associated risks [16,17].

Given these attributes, PEVs hold strong potential as early biomarkers for detecting platelet dysfunction and vascular complications in diabetic patients, both of which are hallmarks of diabetes-related complications [16].

The analysis of PEVs provides a promising non-invasive approach for monitoring platelet function and disease progression in diabetes. Since platelets are directly involved in the pathogenesis of diabetic vascular complications, PEVs reflect molecular changes in the blood coagulation system and vascular health [14,18,19,20]. Recent studies have suggested that changes in the PEV proteome may be used to predict cardiovascular events in diabetic patients [21]. These PEVs can be easily isolated from blood plasma, and their proteomic analysis offers insights into the molecular mechanisms underlying diabetic complications, such as thrombosis, inflammation, and endothelial dysfunction [22]. Therefore, proteomic analysis of PEVs represents a valuable approach for both diagnostic and therapeutic applications in diabetes.

Current methods for studying EVs, including PEVs, often rely on Western blotting; however, this method has limitations in sensitivity and throughput. Mass spectrometry-based approaches, such as matrix-assisted laser desorption/ionization (MALDI) coupled with time-of-flight mass spectrometry (TOF-MS), have gained traction due to their high sensitivity, rapid scan rates, and minimal sample preparation requirements [23,24,25]. MALDI-TOF is particularly well suited for proteomic analyses, as it allows for the detection of intact proteins without the need for extensive sample digestion [26]. It has been successfully applied in bacterial species identification [23], the investigation of phosphorylation sites in EV proteins [25], and the proteomic characterization of microvesicles in various cancers [27,28]. In the context of diabetes, MALDI-TOF offers the potential to detect specific PEV proteins related to platelet activation and thrombotic risk [18]. However, its limitations include ion suppression due to sample contamination, and the necessity of a precise sample-to-matrix ratio for optimal ionization [9,26].

Another promising technology is ion mobility spectrometry (IMS), which separates ions based on their gas-phase mobility. When coupled with time-of-flight mass spectrometry (IMS-TOF), it offers enhanced proteome coverage by separating isobaric species based on their collision cross-section (CCS) [29]. The introduction of trapped ion mobility spectrometry (TIMS) further improves this technique by allowing parallel accumulation and serial fragmentation (PASEF), which eliminates ion loss and increases sensitivity [30,31]. TIMS-TOF has already been applied in diverse proteomic studies, including studies on the analysis of EVs from human and feline plasma [11], and on the proteomics of disease models [32,33]. Additionally, coupling TIMS with nano-liquid chromatography (nanoLC) allows for even greater separation of complex biological samples, such as PEVs, making it an ideal tool for detailed proteomic analysis [11,30].

Given the complementary strengths of MALDI-TOF and TIMS-TOF, we aim to compare nanoLC-MALDI-MS/MS and nanoLC-TIMS-MS/MS for the proteomic analysis of platelet extracellular vesicles (PEVs) in diabetic patients. While alternative approaches, such as DIA-MS and Shotgun Proteomics, were considered, the aforementioned two methods were chosen to maximize analytical coverage and sensitivity.

nanoLC-MALDI-MS/MS was selected for its robustness in detecting intact proteins with minimal sample preparation requirements. Its matrix-assisted ionization process offers enhanced stability, making it particularly suitable for profiling larger proteins and peptides. This technique is also effective for identifying post-translational modifications, which are relevant in diabetic pathology [27,34].

nanoLC-TIMS-MS/MS was chosen for its superior sensitivity and ability to resolve isobaric species through trapped ion mobility spectrometry (TIMS). TIMS enhances protein separation by adding an additional dimension of analysis—ion mobility—which significantly improves the identification of low-abundance proteins, a common challenge in complex biological samples such as PEVs [35].

By combining these two complementary techniques, we aimed to achieve comprehensive proteome coverage and increase the likelihood of identifying key biomarkers associated with diabetes mellitus.

The primary objective of this study is to evaluate which method provides superior sensitivity, accuracy, and overall performance in the identification and quantification of EV-associated proteins. Focusing on PEVs in diabetic patients, our research seeks to expand the toolkit available for biomarker discovery in diabetes, particularly for the early detection of platelet dysfunction and vascular complications, which are critical components in diabetic pathology.

## 2. Results

**Figure 1:**
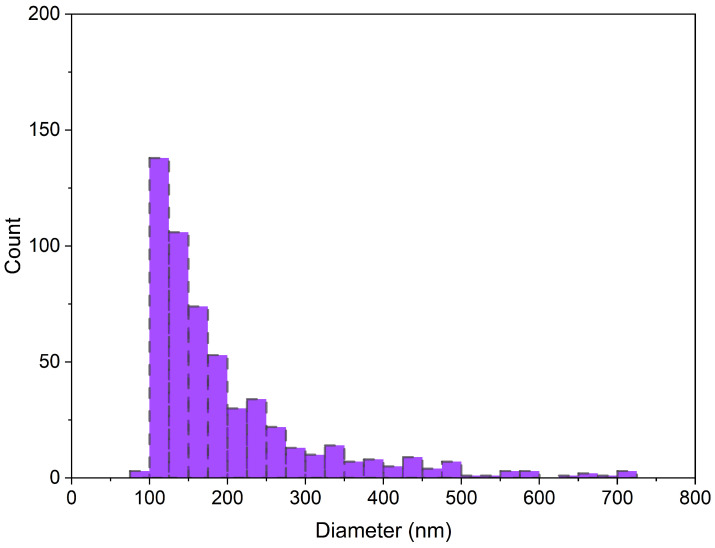
Representative size distribution histogram obtained for extracellular vesicles isolated from platelets (PEVs), analyzed using TRPS technology with IZON qNano system.

In accordance with the guidelines recommended by the International Society for Extracellular Vesicles (ISEV), a quantitative and qualitative analysis of PEV samples was conducted in the initial stage of the study, confirming the presence of EVs in the samples. The size distribution and concentration of EVs isolated from human platelets were analyzed using TRPS technology, allowing for a precise assessment of vesicle size. All measured concentrations were normalized to the initial sample volume. The results showed that the mean number of isolated PEVs was 1.17 × 10^10^ ± 1.11 × 10^9^/mL for the control group and 5.04 × 10^10^ ± 8.50 × 10^9^/mL for diabetics. The analyzed PEVs represented a heterogeneous population of particles, with an average size of 214.83 ± 12.61 nm for the control group and 208.0 ± 17.50 nm for diabetics (Figure 1), and a mode of 129.83 ± 11.13 nm for the control group and 120.50 ± 5.89 nm for diabetics. No significant differences were observed between the groups.

In the analyzed samples, PEVs are present within a particle size range of 80 to 600 nm, which falls within the typical size range of extracellular vesicles that is characteristic of exosome and ectosome fractions (Figure 1).

FTIR spectroscopy identifies biochemical components in biological samples, such as nucleic acids, proteins, lipids, and carbohydrates, by detecting molecular conformations and functional groups. Unique spectral fingerprints reflect genomic, lipidomic, proteomic, and metabolomic parameters, offering insights into disease-specific changes. Key regions include the fingerprint (1450–600 cm^−1^), amide I and II (1700–1500 cm^−1^), and stretching vibrations (3500–2550 cm^−1^) [36].

**Figure 2:**
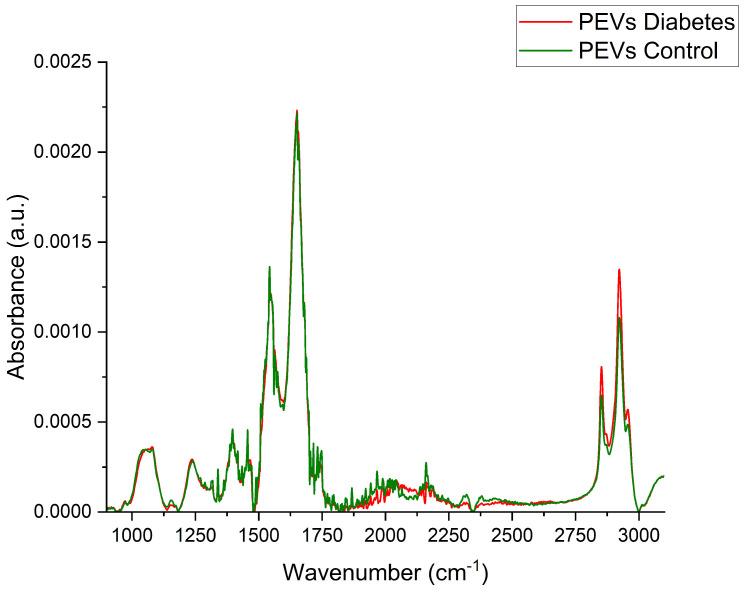
Representative Fourier-transform infrared (FT-IR) spectroscopy spectra derived from PEVs. Spectrum of control group (green line), spectrum of study group (red line).

Figure 2 presents representative FT-IR spectra derived from human PEVs from the control and study groups, while a detailed description of the characteristic IR bands is provided in Table 1.

The obtained PEV spectra exhibited absorption bands of amide I located at 1650 cm^−1^, associated with the stretching mode of the C=O peptide bond, and of amide II located at 1543 cm^−1^, which can be primarily attributed to N-H peptide group vibrations. Also evaluated was the band at 1397 cm^−1^, which can be attributed to symmetric and asymmetric vibrations of COO^−^ groups (1338, 1339) and vibrations of acyl groups at 2922 and 2852 cm^−1^.

**Figure 3:**
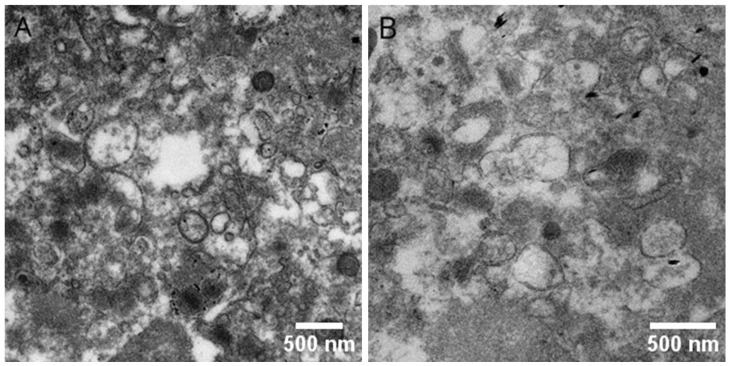
Representative transmission electron microscopy (TEM) images of platelet-derived extracellular vesicles (PEVs) in control group (**A**) and diabetic individuals (**B**).

Transmission electron microscopy (TEM) visualization was used to screen the morphology and size of EV subpopulations derived from human platelets. Representative images of PEVs are presented below (Figure 3). Extracellular vesicles were clearly visible during characterization, forming a heterogeneous mixture of large and small vesicles. Their diameter varied significantly (ranging from 60 nm to 600 nm). All images shown in Figure 3 were obtained using fresh samples. Dense staining around some vesicles indicates the presence of contaminants introduced during the staining process. The obtained results supplemented the data acquired through the TRPS method.

Global research indicates that extracellular vesicles (EVs) can carry bioactive molecules, such as proteins, receptors, mRNA, miRNA, and lipids [37,38,39,40,41,42,43]. To determine the biological content transported by EVs, a global proteomic analysis of human PEV samples was conducted, comparing the application of the nLC-MALDI-TOF-MS/MS method with nLC-TIMS-TOF-MS/MS.

**Figure 4:**
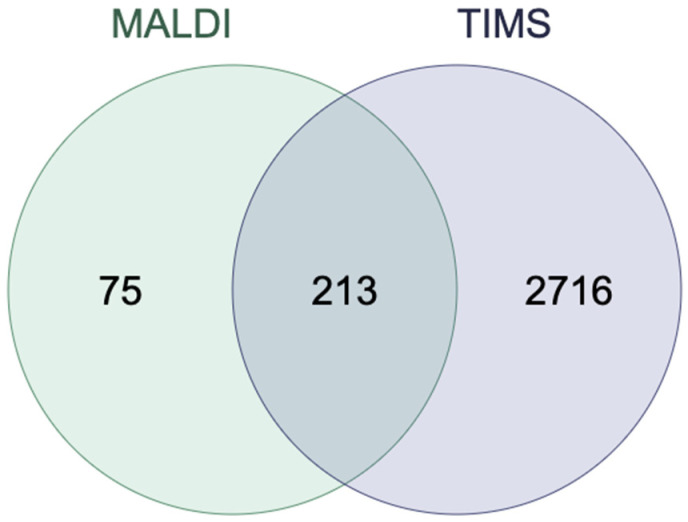
Venn diagram illustrating comparison of proteins identified by matrix-assisted laser desorption/ionization (MALDI) and trapped ion mobility spectrometry (TIMS) in platelet extracellular vesicles (PEVs).

A Venn diagram was used to compare the identification of proteins in extracellular vesicle samples isolated from human platelets carried out using the MALDI and TIMS mass spectrometry methods. A total of 213 common proteins were identified, along with 75 proteins unique to MALDI and 2,716 unique to TIMS. TIMS identified approximately 97% more unique proteins than MALDI (Figure 4). All proteins were identified based on at least two unique peptides.

**Figure 5:**
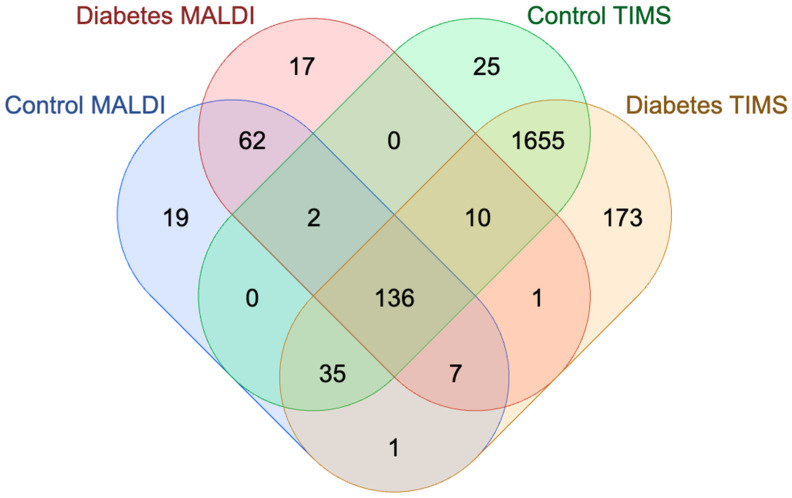
Venn diagram illustrating comparison of proteins identified by matrix-assisted laser desorption/ionization (MALDI) and trapped ion mobility spectrometry (TIMS) in platelet extracellular vesicles (PEVs), divided into control and study (diabetes) groups.

Table 2 presents the number of identified proteins and peptides in the PEV samples analyzed using nLC-MALDI-TOF-MS/MS, both with and without gender differentiation. The Venn diagram (Figure 5) compares the results from the nLC-MALDI-TOF-MS/MS technique with those from nLC-TIMS-TOF-MS/MS for the control and study groups. The highest number of proteins, without gender differentiation, was identified in the control group. In contrast, when analyzing protein identification by gender, more proteins were identified in women than in men. These differences may result from varying levels of gene and protein expression between women and men. Additionally, physiological and metabolic differences between genders could influence protein metabolism. These disparities might also be related to the age and health status of the study subjects. Among diabetic individuals, both without and with gender differentiation, fewer proteins and peptides were identified compared to in the control group.

The study identified 105 out of 304 proteins in human platelets using the nLC-MALDI-TOF-MS technique. A complete list of EV proteins is compiled in Table S1. The nLC-TIMS-TOF-MS technique identified 304 human PEV proteins. A complete list of EV proteins is compiled in Table S2. Proteins identified in human serum from diabetic patients, based on the study by Riaz [34], include Apolipoprotein E, Apolipoprotein A1, Apolipoprotein A2, Apolipoprotein B, and Cholesteryl Ester Transfer Protein (CETP).

The number of proteins and peptides identified using the nLC-TIMS-TOF-MS/MS method in human PEV samples, both with and without gender differentiation, is summarized in Table 3. The total number of proteins identified without gender differentiation in the control group was approximately 7.5% lower than in the study group. However, when comparing the number of identified peptides, there were 10% more peptides observed in diabetics than in the control group. Analyzing the number of identified proteins with gender differentiation, the difference between the control and diabetic groups averaged approximately 2% in women and 29% in men. Furthermore, more proteins were identified in the study group, and fewer in the control group, for men compared to women.

**Figure 6:**
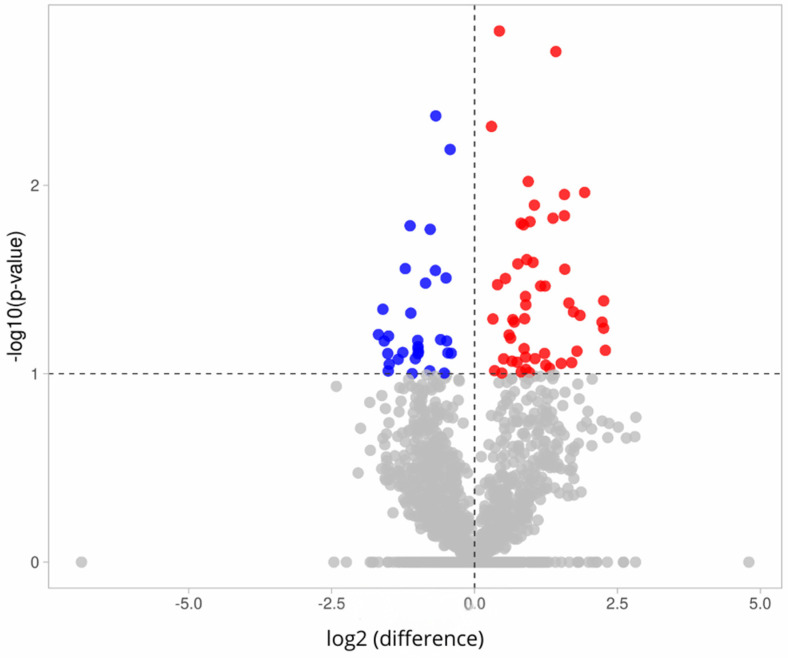
Volcano plot illustrating differential expression of proteins in PEVs between control and study groups among women. X-axis represents fold change (log2(fold change) > 0 indicates increased expression, while log2(fold change) < 0 indicates decreased expression between two groups). Y-axis represents transformation of *p*-values (probability values) into logarithmic scale. Red dots indicate upregulated proteins, i.e., those with increased expression. Blue dots represent downregulated proteins, i.e., those with decreased expression. Gray dots indicate proteins with non-significant expression changes.

The volcano plot in Figure 6 illustrates the differential expression of proteins in PEVs from women in the control and study groups. This plot visualizes the fold change (x-axis) relative to significance (y-axis) for the 1849 identified proteins. Significance (unadjusted *p*-value) and fold change are, respectively, converted to −Log10 (*p*-value) and Log2 (fold change). The vertical and horizontal dashed lines indicate the fold change cutoff point of 1 and a *p*-value of 0.05, respectively. A total of 52 proteins (red dots, upper-right side of Figure 6) were upregulated in the study group, while 31 proteins (blue dots, upper-left side of Figure 6) were downregulated in the study group, compared to the control group. The volcano plot was generated using the VolcaNoseR application (https://huygens.science.uva.nl/VolcaNoseR/, accessed on 11 May 2023). The most significant increase in protein levels was observed for immunoglobulin lambda constant 2 (IGLC2), which increased by 2.29-fold in the diabetes group, while methanethiol oxidase (SELENBP1) showed the most significant decrease in the diabetes group. Detailed results of the differentially expressed PEV proteins in women with diabetes are provided in Table S3.

**Figure 7:**
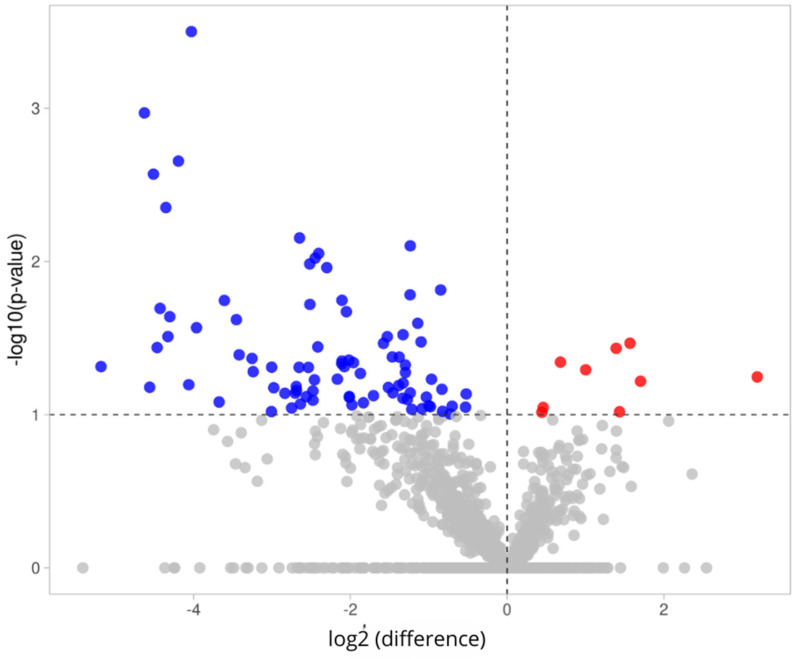
Volcano plot illustrating differences in protein expression in PEVs between control and study groups among men. X-axis represents fold change (log2(fold change) > 0 indicates increased expression, while log2(fold change) < 0 indicates decreased expression between two groups). Y-axis represents transformation of *p*-value (probability values) to logarithmic scale. Red dots indicate upregulated proteins, meaning those with increased expression. Blue dots represent downregulated proteins, meaning those with decreased expression. Gray dots indicate proteins with non-significant expression.

Figure 7 illustrates the differences in protein expression in PEVs between the control and study groups among men. A volcano plot was used to present the 1849 identified proteins. A total of 9 proteins (marked with red dots in the upper-right section) were found to have increased presence in the study group, while 86 proteins (marked with blue dots in the upper-left section) exhibited decreased presence in the study group, compared to the control group. Uromodulin (UMOD) demonstrated the most significant change, increasing by 3.19-fold in the case of diabetes, while envoplakin (EVPL) showed the most substantial decrease in the case of diabetes. The analyzed PEV proteins with differential expression in men with diabetes are provided in Table S4.

**Figure 8:**
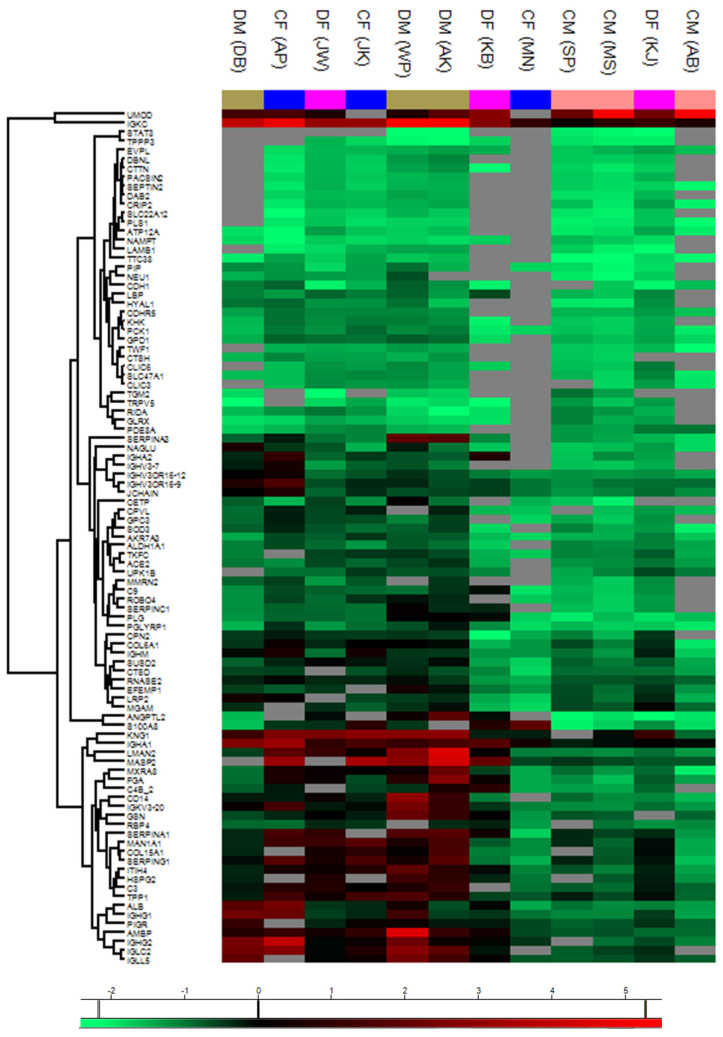
Analysis of gene expression levels in human PEV samples. The gene expression levels were analyzed in human PEV samples across the control groups—CF (control female) and CM (control male)—and the study groups—DF (diabetic female) and DM (diabetic male)—relative to the entire male cohort. The average expression levels within each sample group were calculated based on data obtained from three (N = 3) samples per group. The relative levels of the analyzed genes are presented as a heat map generated using the Perseus tool. The color scale corresponds to the relative mean expression levels in the respective sample types, ranging from relatively lowest (green) to highest (red), as determined by the tool’s scale for a parameter ranging from −2 (green) to 5 (red). The intensity value illustrates the direction of protein expression, categorized as upregulated (increased expression) and downregulated (decreased expression).

Figure 8 presents a heat map of gene expression data from human PEV samples in the control and study groups compared to the overall male cohort. Each row in the heat map represents a gene, while each column corresponds to a specific sample. The normalized LFQ intensity results for 95 PEV genes are shown using a color scale: red indicates positive expression, black denotes equal expression, and green represents negative expression relative to the value of each gene. Detailed information regarding abbreviations is provided in Table S2.

**Figure 9:**
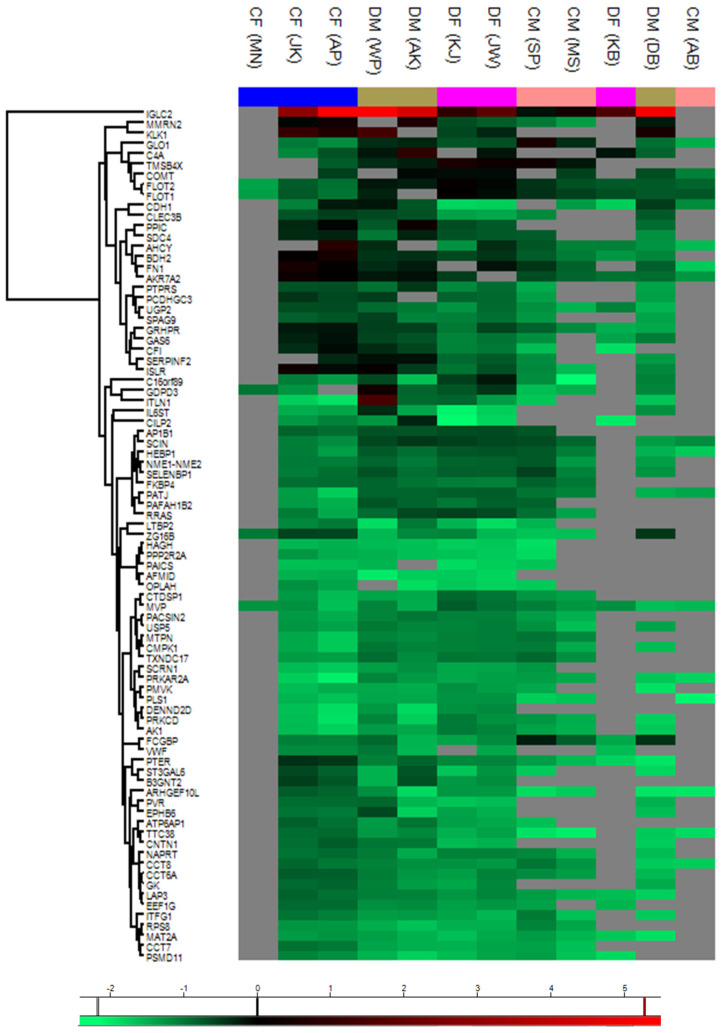
Analysis of gene expression levels in human UEV samples from the control groups, CF (female control) and CM (male control), as well as the study groups, DF (female diabetic) and DM (male diabetic), relative to the entire female group. The average gene expression levels in each sample group were calculated based on data obtained from three (N = 3) samples per group. The relative levels of the analyzed genes are presented as a heat map generated using the Perseus tool. The color scale corresponds to the relative average expression levels in the respective sample types, ranging from the lowest (green) to the highest (red), based on a tool-generated scale with values from −2 (green) to 5 (red). The intensity value illustrates the direction of protein expression as upregulated (increased expression) or downregulated (decreased expression).

Using a heat map (Figure 9), the differences in gene expression in human PEV samples between the control group and the study group are illustrated in relation to the entire female cohort. Each row of the map represents one gene, and each column corresponds to a specific sample. The quantitative intensity results comparing 83 PEV genes (measured as the total MS signal for a given protein) are normalized and visualized using a color scale. Red indicates positive expression, black represents equal expression, and green denotes negative expression compared to the gene’s value. Detailed explanations of abbreviations are provided in Table S4.

## 3. Discussion

This study provides a detailed analysis of platelet extracellular vesicles (PEVs) in diabetic and non-diabetic individuals using advanced analytical techniques, including nanoLC-MALDI-MS/MS, nanoLC-TIMS-MS/MS, TEM, TRPS, and FTIR. By combining these methodologies, we achieved a multidimensional understanding of the morphological, biochemical, and proteomic alterations in PEVs associated with diabetes. Our findings underscore the potential of PEVs as biomarkers for diabetes-related complications and therapeutic targets.

Transmission electron microscopy (TEM) confirmed the extracellular vesicle identity of PEVs by revealing their characteristic double-layered membrane structure. While the average PEV size was consistent across groups, diabetic PEVs exhibited greater size heterogeneity, which may reflect the pathological remodeling of platelets in response to chronic inflammation and oxidative stress in diabetes. This aligns with findings by Freeman et al. [6], which reported similar variability in extracellular vesicle (EV) size in pathological states. Moreover, a recent study by Zhang et al. noted that EV size alterations could serve as indicators of disease progression in metabolic disorders [43].

Tunable resistive pulse sensing (TRPS) further quantified the size distribution and concentration of PEVs, corroborating the morphological findings. Diabetic samples showed a significant increase in PEV concentration, consistent with the hypercoagulable state and chronic platelet activation in diabetes, as noted by Prattichizzo et al. [4]. The elevated concentration and size heterogeneity of PEVs in diabetics highlight the interplay between platelet activation and systemic inflammation in diabetes pathophysiology. Similar observations were reported by López-Canoa et al., who emphasized the role of EVs in coagulation abnormalities and vascular complications [44].

Fourier-transform infrared (FTIR) spectroscopy revealed significant biochemical differences between diabetic and control PEVs. Increased lipid and protein content, as indicated by prominent amide I and II peaks and lipid-associated vibrational bands, were observed in diabetic PEVs. These findings align with Riaz et al. [45], who reported altered lipid metabolism as a hallmark of diabetes. Changes in lipid composition may influence PEV stability, functionality, and interactions with recipient cells. Additionally, Wang et al. (2023) suggested that lipid modifications in EVs play a role in intercellular signaling and inflammatory processes in diabetes [46].

Proteomic analyses using nanoLC-MALDI-MS/MS and nanoLC-TIMS-MS/MS provided comprehensive insights into PEV molecular composition. TIMS demonstrated superior sensitivity, identifying 97% more unique proteins than MALDI, including low-abundance proteins and post-translational modifications. Key proteins identified included apolipoproteins (ApoA1, ApoA2, ApoE) and cholesteryl ester transfer protein (CETP), which were significantly enriched in diabetic PEVs. These proteins play roles in lipid transport and cardiovascular risk, underscoring their relevance in diabetes pathology [31]. Recent work by Dracheva et al. also highlighted the role of apolipoproteins in EV-mediated cholesterol transport and its implications for metabolic syndrome [47].

Additionally, TIMS detected oxidative stress-related proteins such as methanethiol oxidase (SELENBP1), which was significantly downregulated in diabetic PEVs. This aligns with findings by Ye et al. [21], who highlighted the role of SELENBP1 in mitigating oxidative damage. The combined use of MALDI and TIMS underscores their complementary strengths in characterizing PEV proteomes and identifying biomarkers. Quadri et al. demonstrated that TIMS-based proteomic profiling enhances biomarker discovery in EV studies, particularly for metabolic and cardiovascular diseases [48].

Sex-specific analyses revealed distinct proteomic profiles in PEVs from diabetic men and women. In diabetic women, immunoglobulin lambda constant 2 (IGLC2) was significantly upregulated, whereas methanethiol oxidase (SELENBP1) was downregulated. In diabetic men, uromodulin (UMOD) showed the highest upregulation, while envoplakin (EVPL) exhibited the most pronounced downregulation. These findings suggest that sex-specific differences in platelet activation and EV composition may influence disease progression and response to therapy, corroborating studies by Pardo et al. [7]. Recent studies have provided insights into sex-specific differences in extracellular vesicle (EV) profiles, particularly in the context of diabetes. For instance, a study by Diaz Lozano et al. (2022) investigated the proteomic profiles of plasma-derived EVs in non-obese diabetic mice. Their findings revealed distinct protein expression patterns between male and female subjects, suggesting that sex-specific differences in EV composition may influence disease progression and response to therapy. This aligns with observations that certain proteins, such as immunoglobulin lambda constant 2 (IGLC2) and methanethiol oxidase (SELENBP1), exhibit differential expression in diabetic men and women, potentially impacting platelet activation and EV function [49]. Additionally, a systematic review by Arredondo-Damián et al. analyzed the proteome of EVs from the plasma of patients with type 2 diabetes. The study identified several proteins with altered expression levels, implicated in pathophysiological mechanisms such as inflammation and platelet activation. These findings underscore the importance of considering sex-specific differences in EV composition when evaluating disease mechanisms and therapeutic responses in diabetic patients [50]. Furthermore, research by Diaz Lozano et al. demonstrated that proteome profiling of plasma-derived EVs facilitated the detection of tissue biomarkers in non-obese diabetic mice. This study highlights the potential of EVs as carriers of sex-specific biomarkers, which could be pivotal in understanding the differences in disease progression and therapeutic responses between male and female diabetic patients [49]. Collectively, these studies corroborate the notion that sex-specific differences in EV composition and proteomic profiles play a significant role in the pathophysiology of diabetes, influencing both disease progression and response to therapy.

Heat maps provided a visual representation of relative protein expression levels, identifying 95 significantly altered proteins across groups. The integration of bioinformatics tools such as Perseus and VolcaNoseR facilitated the identification of pathways implicated in lipid dysregulation, inflammation, and oxidative stress. Upregulation of apolipoproteins in diabetic PEVs further emphasized their role in metabolic and cardiovascular complications.

The integration of TEM, TRPS, FTIR, MALDI, and TIMS results revealed a consistent pattern of PEV enrichment and functional reprogramming in diabetes. The morphological changes observed in TEM and TRPS complemented the biochemical and proteomic alterations identified by FTIR and mass spectrometry, providing a holistic understanding of PEV dynamics in diabetes. These findings align with a growing body of literature highlighting the potential of EVs as biomarkers and mediators in metabolic diseases [6,45,51,52]. Recent evidence from Zhao et al. further supports the role of EVs in regulating metabolic and inflammatory pathways in diabetes [53].

Additionally, this study highlights the importance of considering sex as a biological variable in diabetes research. The distinct proteomic profiles observed between men and women suggest differential mechanisms of disease progression and therapeutic response.

Recent evidence suggests that sex-specific differences in protein expression may significantly influence these mechanisms, particularly in metabolic disorders such as diabetes. One notable example is asprosin, a fasting-induced hormone encoded by the *FBN1* gene, which demonstrates sex-specific variations in its levels and activity. Asprosin is primarily secreted by white adipose tissue, and plays a key role in glucose release and appetite regulation. Studies have shown that its dysregulation is linked to metabolic disturbances such as obesity and diabetes, with evidence indicating that its expression and function may vary between males and females [54]. These differences highlight the need to consider sex-specific factors when exploring asprosin-targeted therapies.

Another key factor is the G protein-coupled estrogen receptor (GPER), known for mediating estrogen’s rapid effects on various tissues, including the cardiovascular and metabolic systems. The GPER has been implicated in modulating insulin sensitivity, lipid metabolism, and inflammatory pathways, all of which are crucial in diabetes pathophysiology. Importantly, studies indicate that GPER expression and activity may differ between sexes, potentially influencing the efficacy of treatments targeting this pathway [55].

In the context of platelet extracellular vesicles (PEVs), these sex-specific differences in protein profiles may have profound implications for biomarker discovery and therapeutic interventions. The distinct molecular cargo carried by PEVs in males and females could influence disease progression, particularly in conditions such as vascular complications and platelet dysfunction that are commonly observed in diabetic patients [56].

Incorporating these insights into future research may enable the development of personalized therapeutic strategies that account for sex-specific variations in protein expression and function, ultimately improving clinical outcomes in diabetic patients.

Recent studies have refined our understanding of EVs in metabolic diseases. A meta-analysis identified 1717 EV-enriched proteins, providing a valuable resource for biomarker studies in diabetes [45]. Proteomic analyses of serum exosomes in gestational diabetes mellitus (GDM) patients revealed 78 significantly altered proteins, emphasizing the role of EVs in complement and coagulation cascades, platelet activation, and lipid metabolism [7]. These findings align with our observations of altered PEV composition in diabetes, highlighting the utility of advanced analytical techniques in biomarker discovery.

While this study provides comprehensive insights, several limitations must be addressed. The relatively small sample size limits the generalizability of our findings, necessitating validation in larger cohorts. Additionally, the functional roles of the identified biomarkers require further experimental validation. Future studies should explore the integration of proteomics with lipidomics and metabolomics to provide a more comprehensive understanding of PEV-mediated pathways in diabetes.

Longitudinal studies are also needed to evaluate the dynamics of PEV alterations over the course of diabetes progression and treatment. The development of targeted therapies or diagnostic tools based on PEV biomarkers could significantly improve disease management. For instance, TIMS-MS/MS’s ability to resolve complex proteomes could facilitate the identification of novel therapeutic targets or monitor treatment efficacy. Moreover, recent advances in single-vesicle analysis technologies may further refine our understanding of PEV heterogeneity and its clinical relevance.

## 4. Materials and Methods

The majority of reagents were purchased from Sigma-Aldrich (St. Louis, MO, USA), unless otherwise specified in the text.

### 4.1. Blood Samples from Patients

The study was approved by the Bioethics Committee of the Jagiellonian University in Kraków on 27 February 2020 (No. 1072.6120.43.2020). The Committee approved all project protocols and related documentation, including patient information and consent forms. The study group consisted of patients diagnosed with type II diabetes, selected based on clinical data regarding the clinical course and treatment of diabetes. Diabetic patients were treated with medications such as Metformin. The exclusion criteria for the study included the presence or suspicion of a recent bacterial or viral infection; chronic diseases such as diagnosed antiphospholipid syndrome and rheumatoid arthritis; mental illness; kidney disease requiring dialysis; liver disease, such as cirrhosis or hepatitis, under active treatment; elevated CRP levels (>10 mg/L), indicating active inflammation; a history of heart attack, stroke, or surgical operations performed within the last six months; morbid obesity (BMI > 40 kg/m²); a history of bariatric surgery; and active neoplastic disease. The control group consisted of healthy asymptomatic men and women aged 18–85 years. A total of 12 individuals participated in the study, including six participants in the control group (three women and three men) and six participants in the study group (three women and three men). The control group was matched with the study group in terms of gender, age, and race to ensure comparable demographic characteristics.

### 4.2. Sample Collection and PEV Isolation

**Figure 10:**
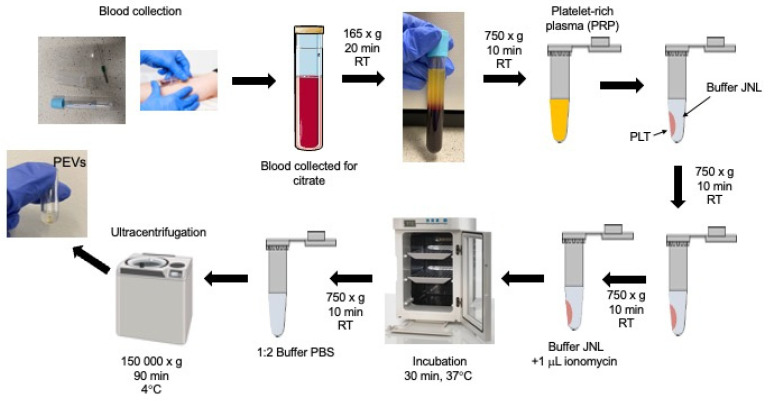
PEV isolation procedure.

Blood samples were collected from 12 donors in the morning at the Diagnostics Department of the University Hospital in Krakow, following a standardized protocol. Samples were drawn into collection tubes containing citrate as an anticoagulant, and then centrifuged at room temperature for 20 min at 165× *g*, to obtain platelet-rich plasma (PRP). The plasma was carefully transferred to a tube and centrifuged again at room temperature for 10 min at 750× *g*. The supernatant (platelet-poor plasma) was collected using a pipette, and the platelet pellet obtained was washed with 3 mL of JNL buffer (130 mM sodium chloride, 10 mM sodium citrate, 9 mM sodium bicarbonate, 6 mM D-glucose, 0.9 mM magnesium chloride, 0.81 mM potassium dihydrogen phosphate, 10 mM Tris, pH 7.4) through gentle pipetting. This washing and centrifugation process was repeated, and the platelets were activated by suspending them in 0.5 mL of JNL buffer with 1 μL of ionomycin, followed by incubating them for 30 min at 37 °C. After incubation, the samples were centrifuged at 750× *g* for 10 min, and the collected supernatant was diluted 1:2 in PBS buffer. Each sample was divided into two parts and ultracentrifuged at 150,000× *g* for 90 min, at 4 °C, (Optima™ MAX-XP, Beckman Coulter Life Sciences, Indianapolis, IN, USA) using a horizontal rotor (Cat. number 367280, MLS-50 Swinging-Bucked Rotor, Beckman Coulter Life Sciences, Indianapolis, IN, USA). The supernatant was decanted, leaving behind the sediment containing the platelet-derived extracellular vesicle (PEV) fraction, which was then resuspended in MiliQ water and frozen at −80 °C until further use. A schematic representation of the isolation procedure for EVs from platelets is provided in Figure 10.

### 4.3. qNano

The size distribution and concentration of the extracellular vesicles isolated from blood platelets were measured using the qNano Gold system equipped with IZON Science Control Suite v.3.1 software. The system was outfitted with an air-based pressure module, enabling the application of the required pressure range. Calibration of the system was conducted using CPC200 calibration beads, following the manufacturer’s instructions. Before measurement, samples were diluted with phosphate-buffered saline (PBS) within the optimal concentration range. NP150 nanopores ranging in size from 60 to 480 nm were employed for measuring the PEV samples. All measurements were conducted in triplicate, and between successive samples, the upper fluid cell was rinsed multiple times with PBS to eliminate any residual particles. A multi-pressure method was utilized for analysis, within a pressure range of 2 to 6 kPa, with a sample volume of 40 μL. The analysis encompassed PEV samples from both the control group and individuals diagnosed with type II diabetes.

### 4.4. Transmission Electron Microscopy (TEM)

To visualize EVs isolated from blood platelets, transmission electron microscopy (TEM) analysis was employed. The EV sediment formed after centrifugation was fixed in 2.5% glutaraldehyde, followed by 0.1 M cacodylate buffer, for 2 h at room temperature. Subsequently, EV fixation was carried out for 1 h in a 1% osmium tetroxide solution. Samples were dehydrated by passing through a series of ethanol concentrations, and were then embedded in PolyBed 812 epoxy resin (Polyscience, Inc., Warrington, PA, USA) at 68 °C. Ultrathin sections were placed on 300-mesh copper grids. Sections containing embedded EVs were stained using uranyl acetate and lead citrate. Observation was performed using a JOEL JEM 2100HT electron microscope (Joel Ltd., Tokyo, Japan), at an accelerating voltage of 80 kV.

### 4.5. Fourier-Transform Infrared Spectroscopy (FTIR)

FTIR spectra of extracellular vesicles derived from blood platelets were collected in the mid-infrared range (4000–400 cm^−1^) using a Nicolet 6700 spectrometer (Thermo Scientific, Rockford, IL, USA) equipped with an Attenuated Total Reflection (ATR) accessory featuring a diamond crystal. A 5 µL EV sample was deposited on the accessory and allowed to dry. Spectra measurements were performed at room temperature, acquiring 256 scans with a nominal resolution of 4 cm^−1^. Subsequently, the spectra underwent initial processing using Spectragryph software version 1.2.15 (Spectroscopy Ninja, Oberstdorf, Germany), which involved baseline correction and smoothing (Savitzky–Golay filter, second-order polynomial).

### 4.6. Protein Quantification

The total protein content of the platelet-derived extracellular vesicle pellets was measured using the Pierce BCA Protein Assay Kit (Thermo Scientific, Rockford, IL, USA), according to the manufacturer’s instructions, with bovine serum albumin (BSA) as the standard. Absorbance measurements were performed at 595 nm with an Infinite 200 Pro spectrophotometer (Tecan Trading AG, Switzerland), with three replicates conducted for each sample. The average protein concentration ranged between 0.06 and 0.90 mg/mL. A total protein amount of 10 μg was utilized for mass spectrometry analysis.

### 4.7. Protein Digestion

A volume of 10 µL of the isolated sample was combined with 1.5 µL of 100 mM dithiothreitol (DTT), 15 µL of 50 mM ammonium bicarbonate (AMBIC), and 0.5 µL of deionized water. The mixture was incubated for 5 min at 95 °C, and subsequently cooled to room temperature. Following cooling, 3 µL of 100 mM iodoacetamide (IAA) was added to the sample, which was then incubated at room temperature in the dark for 20 min. Protein digestion was carried out using MS-grade trypsin gold (Promega, UK) at 37 °C, with an enzyme-to-protein ratio of 1:50. To ensure complete digestion, an additional aliquot of trypsin (1:100 enzyme-to-protein ratio) was added after 3 h, and digestion continued overnight at 37 °C. Acidification of the sample was achieved by adding 1.5 µL of 10% trifluoroacetic acid (TFA).

The resulting peptides were purified using two C18 minicolumns, each containing 6 plugs of C18 extraction disk, following a modified procedure based on Wiśniewski [57]. Subsequently, all peptide fractions were dried using a CentriVap (Labconco, Kansas City, MO, USA), and resuspended in 60 µL of 0.1% trifluoroacetic acid (TFA), before being frozen at −20 °C until instrumental analysis.

### 4.8. Proteomic Analysis of Platelets Vesicles by nanoLC-MALDI-MS/MS

The chromatographic separation of peptides was carried out using nanoLC in a reverse-phase mode with fraction spotting on a MALDI plate. A stainless steel MTP AnchorChip™ 800/384 TF plate was placed in one of the four positions of the PROTEINEER fc II fraction collector. Alpha-cyano-4-hydroxycinnamic acid (HCCA) was used as the matrix. In a vial, a saturated matrix solution was prepared from approximately 10 mg of HCCA, to which 180 μL of ACN, 18 μL of H_2_O, and 2 μL of 10% TFA were added. The mixture was sonicated in an ultrasonic bath for 10 min, followed by centrifugation at 14,000× *g* for 10 min. Matrix solutions for the sample and calibration solution were prepared as follows. For each of two 1.5 mL Eppendorf tubes, 72 μL of saturated HCCA solution, 8 μL of 10% TFA, 8 μL of 100 mM NH_4_H_2_PO_4_, and 721 μL of TA95 (95:5 (*v*/*v*) ACN:0.1% TFA) were added to create the HCCA solution for the samples, and 721 μL of TA85 (85:5 (*v/v*) ACN:0.1% TFA) were added to create the HCCA solution for the calibration solution. A Hamilton I/D 4.61 mm, 1000 μL syringe was mounted in the fraction collector, filled with 400 μL of the working matrix solution for samples, for internal pumping. Chromatographic separation was performed using the EASY-nLC II™ nano liquid chromatograph. A gradient elution from 2% to 45% of solvent B over 80 min, followed by a sharp increase to 100% of solvent B within 0.1 min, was implemented. The column temperature was controlled at 7 °C, and the flow rate was maintained at 300 nL min^−1^. The chromatographic separation was carried out on an Acclaim^®^ PepMap 100 chromatographic column (75 μm × 15 cm C18, 3 μm, 100 Å). The sample was conditioned for 10 min before introduction into the analytical column [sample buffer flow rate (0.1% TFA) 500 nL/min] using the Acclaim^®^ PepMap 100 precolumn (100 μm × 2 cm C18, 5 μm, 100 Å). After the sample separation was completed, 0.5 μL of a calibration solution, consisting of 100 μL of HCCA solution, and 0.5 μL of Peptide Calibration Standard II were applied to the appropriate spots on the MALDI plate. The plate was placed in the ultrafleXtreme™ mass spectrometer.

The MALDI-TOF/TOF ultrafleXtreme system was used for analysis, equipped with a 337 nm wavelength laser and operated by the flexControl 3.3 build 108 system. Prior to measurements, the mass spectrometer was calibrated using peptide standards (Peptide Calibration Standards II) in the mass range of 680–4000 Da. The spectroscopic analysis was performed in data-dependent automated mode. The MS and MS/MS parameters were set as follows: ions were accelerated at 25 kV with delayed extraction of 120 ns, ion source 2: 22.35 kV, lens: 7.60 kV, reflector 1: 26.45 kV, reflector 2: 13.40 kV, detection gain: 16.3, sampling rate: 1.00 GS/s. Each MS spectrum was composed of 3500 laser shots, and for the MS/MS spectrum, 5000 laser shots were accumulated. The acquired mass spectra were analyzed using Flex Analysis 3.3 build 80 software. Non-redundant precursor peptides were selected for MS/MS using WARP-LC 1.2 software with a signal-to-noise threshold of 10. Protein identification was performed using BioTools 3.2 Build 6.32 software and Matrix Science MASCOT Server 2.4 (in-house) (http://www.matrixscience.com (accessed on 3 January 2025), Matrix Science Ltd., London, UK), utilizing protein and nucleotide databases such as NCBI and SwissProt. The general search parameters were as follows: species: Homo sapiens, enzyme: trypsin, protein chain modifications: carbamidomethylation (fixed), oxidation (variable), and N-terminal acetylation (variable), peptide precursor mass tolerance: 50 ppm, fragment mass tolerance: 0.2 Da, charge: +1, mono-isotopic mass.

### 4.9. Proteomic Analysis of Platelet Vesicles by nanoLC-TIMS-MS/MS

Prior to nanoLC-MS/MS analysis, an additional round of peptide desalting was performed, involving 2 µg of peptides. This process was carried out using SPE (Solid Phase Extraction) with Pierce C18 spin columns from Thermo Scientific, following the manufacturer’s protocol. The desalted peptides were then dried and reconstituted in a solution of 20 μL containing 2% acetonitrile (ACN) and 0.1% formic acid (Sigma-Aldrich, Hamburg, Germany). Subsequently, 1 µL of the peptide digest was introduced into the Ultimate3000 nanocapillary chromatography system from Thermo Scientific. The separations were executed with an Aurora column (25 cm in length, 75 μm internal diameter, C18, IonOptics), in conjunction with a precolumn PepMap100 C18 5 µm (5 mm long, 0.3 mm internal diameter, Thermo Scientific). The chromatographic gradient was generated using two solvents: solvent A, consisting of 0.1% formic acid in water (H_2_O), and solvent B, composed of 0.1% formic acid in acetonitrile (ACN). The flow rate was set at 300 nL/min, and the gradient was initiated at 2% B, ramping up to 35% B over 90 min, followed by an isocratic hold at 85% B for 7 min. The entire chromatographic system was managed using Hystar software provided by Bruker Daltonics. The chromatography system was directly connected to the timsTOF Pro 2 mass spectrometer, also from Bruker Daltonics, which operated in a positive-ion mode. The mass spectrometry scans spanned a range from 100 to 1700 *m*/*z*, and the ion mobility covered a 1/K0 range from 0.6 to 1.6. Fragmentation of ions was achieved utilizing the parallel accumulation and serial fragmentation (PASEF) method, with standard settings. The acquired mass spectra and chromatograms were analyzed using FragPipe software version 19.0, employing the Human Proteome database with the ID UP000005640 from Uniprot. The search parameters were configured as follows: taxonomy: Homo sapiens (Human); modifications: carbamidomethylation of C (fixed), oxidation of M (variable), and acetylation of N-terminus (variable), allowing for up to 2 missed cleavages. Peptide charges of +2, +3, and +4 were considered, and the mass tolerance was set at 20 ppm for both MS and MS/MS. All other parameters were maintained as the default settings for TIMS with PASEF data.

### 4.10. Bioinformatic Analysis

The acquired mass spectra and chromatograms were analyzed using FragPipe version 19.1 and ProteinScape to perform protein identification and determine protein accumulation. Prior to each data analysis, normalization and transformation (logarithmization) were conducted on the corresponding dataset using the Perseus platform version 1.6.5.0.

Classification of proteins based on their origin, function, and biological processes was carried out using the PANTHER 17.0 classification system and FunRich version 3.1.4. Comparisons between the studied groups were visualized using Venn diagrams, facilitated by the Multiple List Comparator (www.molbiotools.com/listcompare.html (accessed on 3 January 2025)).

Differential quantitative analysis of human and rat UEV and PEV proteins was presented in the form of volcano plots and heat maps. The obtained results were analyzed using the Perseus platform version 1.6.5.0 and VolcaNoseR (http://huygens.science.uva.nl/VolcaNoseR (accessed on 3 January 2025)). To identify differential proteins between the study and control groups, Student’s t-test with a false discovery rate (FDR) set at 1% was applied. Only proteins with at least two unique peptides and a change value threshold above 1 were considered. The color scale on the heat map charts illustrates the relative protein expression levels by comparing control samples to diabetic ones.

## 5. Conclusions

This study demonstrates the utility of advanced analytical techniques in characterizing PEVs in diabetes. By leveraging the strengths of nanoLC-TIMS-MS/MS, we identified critical biomarkers and pathways that may inform future diagnostic and therapeutic strategies. These findings advance our understanding of PEV biology and highlight the importance of tailoring analytical approaches to specific biological questions.

## Figures and Tables

**Table 1:** Characteristic IR bands analyzed in human PEV samples.

Wavenumber of PEVs (Control) (cm^−1^)	Wavenumber of PEVs (Diabetes) (cm^−1^)	Band Assignment
1154, 1313, 1397,1543, 1650	1156, 1339, 1396, 1543, 1650	Proteins
1456, 2852, 2922	1456, 2852, 2922	Lipids
1080, 1239	1078, 1242	Nucleic acids
970	970	Carbohydrates

**Table 2:** Number of identified proteins and peptides in human PEV samples using nLC-MALDI-TOF-MS/MS technique.

		Group	Number of Identified Proteins	Number of Identified Peptides
	
Total (no gender division)		Control	259	2445
		Diabetes	233	2588
Women		Control	224	1896
		Diabetes	154	1487
Men		Control	216	1923
		Diabetes	207	1617

**Table 3:** Number of identified proteins and peptides in human PEV samples using nLC-TIMS-TOF-MS/MS technique.

		Group	Number of Identified Proteins	Number of Identified Peptides
	
Total (no gender division)		Control	1863	17,828
		Diabetes	2015	19,895
Women		Control	1778	16,785
		Diabetes	1813	17,428
Men		Control	1376	11,892
		Diabetes	207	1617

## Data Availability

The authors confirm that most of the data supporting the findings of this study are available within the article and its Supplementary Materials. The raw data are available from the corresponding author (J.K.-P.) upon request.

## Supplementary Materials

The following supporting information can be downloaded at https://www.mdpi.com/article/10.3390/molecules30061384/s1, Table S1: List of human PEV proteins derived from ExoCarta (exosome database). Exosomal proteins identified in human PEVs using nLC-MALDI-TOF-MS/MS technique; Table S2: List of human PEV proteins derived from ExoCarta (exosome database). Exosomal proteins identified in human PEVs using nLC-TIMS-TOF-MS/MS technique; Table S3: List of analyzed PEV proteins with differential expression in women with diabetes (Figure 6); Table S4: List of analyzed PEV proteins with differential expression in men with diabetes (Figure 7); List S1: Expansion of protein abbreviations presented on heat map in Figure 8; List S2: Expansion of protein abbreviations presented on heat map in Figure 9.
