# Supplementary material for: The Proteomic Analysis of Platelet Extracellular Vesicles in Diabetic Patients by nanoLC-MALDI-MS/MS and nanoLC-TIMS-MS/MS
Source: Molecules. 2025 Mar 20;30(6):1384. doi: 10.3390/molecules30061384 (PMC11944696; doi:10.3390/molecules30061384)
Supplement: Supplementary file 1 [file molecules-30-01384-s001.zip › Supplementary files/List S1.pdf]

List S1: Expansion of protein abbreviations presented on the heat map in Figure 8.

UMOD – Uromodulin
RIDA – Reactive Intermediate Deaminase A
TGM2 – Transglutaminase 2
TRPV5 – Transient Receptor Potential Cation Channel Subfamily V Member 5
GLRX – Glutaredoxin
PDE8A – Phosphodiesterase 8A
STAT3 – Signal Transducer and Activator of Transcription 3
NAMPT – Nicotinamide Phosphoribosyltransferase
TPPP3 – Tubulin Polymerization-Promoting Protein Family Member 3
EVPL – Envoplakin
ATP12A – ATPase H+/K+ Transporting Subunit Alpha 2
PACSIN2 – Protein Kinase C and Casein Kinase Substrate in Neurons Protein 2
CTSH – Cathepsin H
PLS1 – Plastin 1
ALDH1A1 – Aldehyde Dehydrogenase 1 Family Member A1
CDHR5 – Cadherin-Related Family Member 5
SLC22A12 – Solute Carrier Family 22 Member 12
CLIC6 – Chloride Intracellular Channel 6
SLC47A1 – Solute Carrier Family 47 Member 1
SEPTIN2 – Septin 2
CRIP2 – Cysteine-Rich Protein 2
TTC38 – Tetratricopeptide Repeat Domain 38
MGAM – Maltase-Glucoamylase
KHK – Ketohexokinase
DAB2 – Disabled-2
LAMB1 – Laminin Subunit Beta 1
RNASE2 – Ribonuclease A Family Member 2
TWF1 – Twinfilin Actin Binding Protein 1
CLIC3 – Chloride Intracellular Channel 3
UPK1B – Uroplakin 1B
PCK1 – Phosphoenolpyruvate Carboxykinase 1
DBNL – Drebrin-Like Protein
TKFC – Triokinase and FMN Cyclase
CTSD – Cathepsin D
LBP – Lipopolysaccharide Binding Protein
AKR7A3 – Aldo-Keto Reductase Family 7 Member A3
CTTN – Cortactin
GPD1 – Glycerol-3-Phosphate Dehydrogenase 1
ACE2 – Angiotensin-Converting Enzyme 2
CDH1 – Cadherin-1 (E-Cadherin)
SUSD2 – Sushi Domain Containing 2
RBP4 – Retinol Binding Protein 4
GPC3 – Glypican 3
PIP – Prolactin Induced Protein
C9 – Complement Component 9
LRP2 – Low Density Lipoprotein Receptor-Related Protein 2
HYAL1 – Hyaluronidase 1
EFEMP1 – EGF Containing Fibulin Extracellular Matrix Protein 1
JCHAIN – Joining Chain of Multimeric IgA and IgM

IGHV3OR16-9 – Immunoglobulin Heavy Variable 3-OR16-9
IGHA2 – Immunoglobulin Heavy Constant Alpha 2
CPN2 – Carboxypeptidase N Subunit 2
SOD3 – Superoxide Dismutase 3
TPP1 – Tripeptidyl Peptidase 1
IGHA1 – Immunoglobulin Heavy Constant Alpha 1
PIGR – Polymeric Immunoglobulin Receptor
CPVL – Carboxypeptidase Vitellogenic Like
IGHV3OR16-12 – Immunoglobulin Heavy Variable 3-OR16-12
S100A8 – S100 Calcium Binding Protein A8
PGLYRP1 – Peptidoglycan Recognition Protein 1
HSPG2 – Heparan Sulfate Proteoglycan 2
IGHM – Immunoglobulin Heavy Constant Mu
MMRN2 – Multimerin 2
IGHV3-7 – Immunoglobulin Heavy Variable 3-7
COL15A1 – Collagen Type XV Alpha 1 Chain
GSN – Gelsolin
C3 – Complement Component 3
NEU1 – Neuraminidase 1
MAN1A1 – Mannosidase Alpha Class 1A Member 1
COL6A1 – Collagen Type VI Alpha 1 Chain
C4B_2 – Complement C4B-2
ROBO4 – Roundabout Guidance Receptor 4
SERPINA1 – Serpin Family A Member 1
SERPING1 – Serpin Family G Member 1
KNG1 – Kininogen 1
SERPINC1 – Serpin Family C Member 1
CETP – Cholesteryl Ester Transfer Protein
ALB – Albumin
PLG – Plasminogen
ITIH4 – Inter-Alpha-Trypsin Inhibitor Heavy Chain 4
NAGLU – N-Acetyl-Alpha-Glucosaminidase
MXRA8 – Matrix Remodeling Associated 8
IGHG1 – Immunoglobulin Heavy Constant Gamma 1
IGLL5 – Immunoglobulin Lambda Like Polypeptide 5
FGA – Fibrinogen Alpha Chain
IGLC2 – Immunoglobulin Lambda Constant 2
MASP2 – Mannan-Binding Lectin Serine Peptidase 2
AMBP – Alpha-1-Microglobulin/Bikunin Precursor
IGHG2 – Immunoglobulin Heavy Constant Gamma 2
CD14 – CD14 Molecule
SERPINA3 – Serpin Family A Member 3
IGKV3-20 – Immunoglobulin Kappa Variable 3-20
ANGPTL2 – Angiopoietin-Like 2
IGKC – Immunoglobulin Kappa Constant
LMAN2 – Lectin Mannose Binding 2
