# Supplementary material for: The Proteomic Analysis of Platelet Extracellular Vesicles in Diabetic Patients by nanoLC-MALDI-MS/MS and nanoLC-TIMS-MS/MS
Source: Molecules. 2025 Mar 20;30(6):1384. doi: 10.3390/molecules30061384 (PMC11944696; doi:10.3390/molecules30061384)
Supplement: Supplementary file 1 [file molecules-30-01384-s001.zip › Supplementary files/List S2.pdf]

List S2: Expansion of protein abbreviations presented on the heat map in Figure 9.

IGLC2 – Immunoglobulin Lambda Constant 2
CDH1 – Cadherin 1 (E-Cadherin)
CFI – Complement Factor I
AHCY – Adenosylhomocysteinase
GAS6 – Growth Arrest Specific 6
PTER – Phosphotriesterase-Related
BDH2 – 3-Hydroxybutyrate Dehydrogenase Type 2
ISLR – Immunoglobulin Superfamily Containing Leucine-Rich Repeat
GRHPR – Glyoxylate Reductase and Hydroxypyruvate Reductase
KLK1 – Kallikrein 1
MMRN2 – Multimerin 2
CILP2 – Cartilage Intermediate Layer Protein 2
CLEC3B – C-Type Lectin Domain Family 3 Member B
ST3GAL6 – ST3 Beta-Galactoside Alpha-2,3-Sialyltransferase 6
PVR – Poliovirus Receptor
CNTN1 – Contactin 1
AKR7A2 – Aldo-Keto Reductase Family 7 Member A2
ZG16B – Zymogen Granule Protein 16B
B3GNT2 – Beta-1,3-N-Acetylglucosaminyltransferase 2
LTBP2 – Latent Transforming Growth Factor Beta Binding Protein 2
SERPINF2 – Serpin Family F Member 2 (Alpha-2-Antiplasmin)
PPIC – Peptidylprolyl Isomerase C
TTC38 – Tetratricopeptide Repeat Domain 38
ATP6AP1 – ATPase H+ Transporting Accessory Protein 1
EEF1G – Eukaryotic Translation Elongation Factor 1 Gamma
IL6ST – Interleukin 6 Signal Transducer (GP130)
ARHGEF10L – Rho Guanine Nucleotide Exchange Factor 10 Like
CCT7 – Chaperonin Containing TCP1 Subunit 7
LAP3 – Leucine Aminopeptidase 3
UGP2 – UDP-Glucose Pyrophosphorylase 2
PSMD11 – Proteasome 26S Subunit, Non-ATPase 11
ITFG1 – Integrin Alpha FG-GAP Repeat Containing 1
OPLAH – 5-Oxoprolinase
CCT6A – Chaperonin Containing TCP1 Subunit 6A
EPHB6 – EPH Receptor B6
GK – Glycerol Kinase
SDC4 – Syndecan 4
FN1 – Fibronectin 1
PTPRS – Protein Tyrosine Phosphatase Receptor Type S
PCDHGC3 – Protocadherin Gamma Subfamily C Member 3
VWF – Von Willebrand Factor
PPP2R2A – Protein Phosphatase 2 Regulatory Subunit B Alpha
CCT8 – Chaperonin Containing TCP1 Subunit 8
MAT2A – Methionine Adenosyltransferase 2A
AFMID – Arylformamidase
SPAG9 – Sperm Associated Antigen 9
FCGBP – Fc Fragment of IgG Binding Protein
RPS8 – Ribosomal Protein S8
HAGH – Hydroxyacylglutathione Hydrolase

PAICS – Phosphoribosylaminoimidazole Carboxylase
NAPRT – Nicotinate Phosphoribosyltransferase
FKBP4 – FK506 Binding Protein 4
SELENBP1 – Selenium Binding Protein 1
NME1-NME2 – NME1-NME2 Readthrough Transcript
SCRN1 – Secernin 1
USP5 – Ubiquitin Specific Peptidase 5
AP1B1 – Adaptor Related Protein Complex 1 Beta 1 Subunit
PMVK – Phosphomevalonate Kinase
PACSIN2 – Protein Kinase C and Casein Kinase Substrate in Neurons 2
TXNDC17 – Thioredoxin Domain Containing 17
PLS1 – Plastin 1
COMT – Catechol-O-Methyltransferase
MVP – Major Vault Protein
GLO1 – Glyoxalase 1
CMPK1 – Cytidine/Uridine Monophosphate Kinase 1
HEBP1 – Heme Binding Protein 1
MTPN – Myotrophin
PRKAR2A – Protein Kinase CAMP-Dependent Type II Regulatory Subunit Alpha
PAFAH1B2 – Platelet-Activating Factor Acetylhydrolase 1B Subunit 2
CTDSP1 – CTD Small Phosphatase 1
GDPD3 – Glycerophosphodiester Phosphodiesterase Domain Containing 3
DENND2D – DENN Domain Containing 2D
SCIN – Scinderin
AK1 – Adenylate Kinase 1
PRKCD – Protein Kinase C Delta
RRAS – RAS Related Gene 1
TMSB4X – Thymosin Beta 4 X-Linked
PATJ – Pals1 Associated Tight Junction Protein
ITLN1 – Intelectin 1
C4A – Complement Component 4A
FLOT2 – Flotillin 2
FLOT1 – Flotillin 1
C16orf89 – Chromosome 16 Open Reading Frame 89
