# Supplementary material for: The Proteomic Analysis of Platelet Extracellular Vesicles in Diabetic Patients by nanoLC-MALDI-MS/MS and nanoLC-TIMS-MS/MS
Source: Molecules. 2025 Mar 20;30(6):1384. doi: 10.3390/molecules30061384 (PMC11944696; doi:10.3390/molecules30061384)
Supplement: Supplementary file 1 [file molecules-30-01384-s001.zip › Supplementary files/Table S1.pdf]

Table S1: List of human PEV proteins derived from ExoCarta (exosome database). Exosomal proteins identified in human PEVs using the nLC-MALDI-TOF-MS/MS technique.

Np.	Protein ID	Protein name
1	ACLY	ATP citrate lyase
2	ACTB	actin, beta
3	ACTN1	actinin, alpha 1
4	ACTN4	actinin, alpha 4
5	ALDOA	aldolase A, fructose-bisphosphate
6	ALDOC	aldolase C, fructose-bisphosphate
7	ARF1	ADP-ribosylation factor 1
8	ARF3	ADP-ribosylation factor 3
9	ARPC2	actin related protein 2/3 complex, subunit 2, 34kDa
10	ARPC3	actin related protein 2/3 complex, subunit 3, 21kDa
11	ARPC4	actin related protein 2/3 complex, subunit 4, 20kDa
12	BIN2	bridging integrator 2
13	BLMH	bleomycin hydrolase
14	CAP1	CAP, adenylate cyclase-associated protein 1 (yeast)
15	CAPZB	capping protein (actin filament) muscle Z-line, beta
16	CD109	CD109 molecule
17	CD226	CD226 molecule
18	CD36	CD36 molecule (thrombospondin receptor)
19	CD9	CD9 molecule
20	CDC42	cell division cycle 42
21	CLIC1	chloride intracellular channel 1
22	COTL1	coactosin-like F-actin binding protein 1
23	DBNL	drebrin-like
24	DNM1L	dynamin 1-like
25	EHD3	EH-domain containing 3
26	ERP44	endoplasmic reticulum protein 44
27	ESAM	endothelial cell adhesion molecule
28	ESYT1	extended synaptotagmin-like protein 1
29	FLNA	filamin A, alpha
30	G6PD	glucose-6-phosphate dehydrogenase
31	GANAB	glucosidase, alpha; neutral AB
32	GNAI2	guanine nucleotide binding protein (G protein), alpha inhibiting activity polypeptide 2
33	GNAQ	guanine nucleotide binding protein (G protein), q polypeptide
34	GNAZ	guanine nucleotide binding protein (G protein), alpha z polypeptide
35	GP1BA	glycoprotein Ib (platelet), alpha polypeptide
36	GP1BB	glycoprotein Ib (platelet), beta polypeptide
37	GSTO1	glutathione S-transferase omega 1
38	GSTP1	glutathione S-transferase pi 1
39	HBB	hemoglobin, beta
40	HPSE	heparanase
41	HSPB1	heat shock 27kDa protein 1
42	ICAM2	intercellular adhesion molecule 2
43	IGHA1	immunoglobulin heavy constant alpha 1
44	IGHG1	immunoglobulin heavy constant gamma 1 (G1m marker)
45	IGHG2	immunoglobulin heavy constant gamma 2 (G2m marker)

46	IGHG4	immunoglobulin heavy constant gamma 4 (G4m marker)
47	IGHM	immunoglobulin heavy constant mu
48	IGKC	immunoglobulin kappa constant
49	IGLC3	immunoglobulin lambda constant 3 (Kern-Oz+ marker)
50	ILK	integrin-linked kinase
51	INF2	inverted formin, FH2 and WH2 domain containing
52	LCK	LCK proto-oncogene, Src family tyrosine kinase
53	LDHB	lactate dehydrogenase B
54	LIMS1	LIM and senescent cell antigen-like domains 1
55	LRBA	LPS-responsive vesicle trafficking, beach and anchor containing
56	LTBP1	latent transforming growth factor beta binding protein 1
57	MGLL	monoglyceride lipase
58	MMRN1	multimerin 1
59	MTPN	myotrophin
60	MYH9	myosin, heavy chain 9, non-muscle
61	MYL6	myosin, light chain 6, alkali, smooth muscle and non-muscle
62	MYL9	myosin, light chain 9, regulatory
63	PARVB	parvin, beta
64	PDIA3	protein disulfide isomerase family A, member 3
65	PDIA6	protein disulfide isomerase family A, member 6
66	PEAR1	platelet endothelial aggregation receptor 1
67	PGAM1	phosphoglycerate mutase 1 (brain)
68	PGK1	phosphoglycerate kinase 1
69	PGM1	phosphoglucomutase 1
70	PLEK	pleckstrin
71	PPIA	peptidylprolyl isomerase A (cyclophilin A)
72	PPIB	peptidylprolyl isomerase B (cyclophilin B)
73	PRDX4	peroxiredoxin 4
74	PRDX6	peroxiredoxin 6
75	PTPRJ	protein tyrosine phosphatase, receptor type, J
76	PYGB	phosphorylase, glycogen; brain
77	PYGL	phosphorylase, glycogen, liver
78	RAB10	RAB10, member RAS oncogene family
79	RAB14	RAB14, member RAS oncogene family
80	RAB1C	RAB1C, member RAS oncogene family pseudogene
81	RAB7A	RAB7A, member RAS oncogene family
82	RAB8A	RAB8A, member RAS oncogene family
83	RAC3	ras-related C3 botulinum toxin substrate 3 (rho family, small GTP binding protein Rac3)
84	RALB	v-ral simian leukemia viral oncogene homolog B
85	RAP1B	RAP1B, member of RAS oncogene family
86	RAP2B	RAP2B, member of RAS oncogene family
87	RASA3	RAS p21 protein activator 3
88	RHOA	ras homolog family member A
89	RSU1	Ras suppressor protein 1
90	RTN4	reticulon 4
91	SEPT6	septin 6
92	SRC	SRC proto-oncogene, non-receptor tyrosine kinase
93	STOM	stomatin
94	STX11	syntaxin 11

95	TGFB1	transforming growth factor, beta 1
96	TLN1	talin 1
97	TPM3	tropomyosin 3
98	TPM4	tropomyosin 4
99	TTYH3	tweety family member 3
100	UBA1	ubiquitin-like modifier activating enzyme 1
101	UBA7	ubiquitin-like modifier activating enzyme 7
102	VASP	vasodilator-stimulated phosphoprotein
103	VWF	von Willebrand factor
104	WDR1	WD repeat domain 1
105	ZYX	zyxin
