# Supplementary material for: The Proteomic Analysis of Platelet Extracellular Vesicles in Diabetic Patients by nanoLC-MALDI-MS/MS and nanoLC-TIMS-MS/MS
Source: Molecules. 2025 Mar 20;30(6):1384. doi: 10.3390/molecules30061384 (PMC11944696; doi:10.3390/molecules30061384)
Supplement: Supplementary file 1 [file molecules-30-01384-s001.zip › Supplementary files/Table S2.pdf]

Table S2: List of human PEV proteins derived from ExoCarta (exosome database). Exosomal proteins identified in human PEVs using the nLC-TIMS-TOF-MS/MS technique.

No.	Protein ID	Protein Name
1	ABCB6	ATP-binding cassette, subfamily B (MDR/TAP), member 6 (Langereis blood group)
2	ABI1	ABL interactor 1
3	ACLY	ATP citrate lyase
4	ACSL4	Long-chain acyl-CoA synthetase family member 4
5	ACTB	Actin, beta
6	ACTN1	Actinin, alpha 1
7	ACTN4	Actinin, alpha 4
8	ACVR1	Activin A receptor type I
9	ADCY6	Adenylate cyclase 6
10	ALDOA	Aldolase A, fructose-bisphosphate
11	ALDOC	Aldolase C, fructose-bisphosphate
12	ANO6	Anoctamin 6
13	ANXA1	Annexin A1
14	ANXA2	Annexin A2
15	ANXA3	Annexin A3
16	ANXA4	Annexin A4
17	ANXA5	Annexin A5
18	ANXA6	Annexin A6
19	ANXA7	Annexin A7
20	AP1B1	Adaptor-related protein complex 1, beta 1 subunit
21	AP1G1	Adaptor-related protein complex 1, gamma 1 subunit
22	AP2A1	Adaptor-related protein complex 2, alpha 1 subunit
23	AP2B1	Adaptor-related protein complex 2, beta 1 subunit
24	APOA1	Apolipoprotein A-I
25	APOA2	Apolipoprotein A-II
26	APOA4	Apolipoprotein A-IV
27	APOB	Apolipoprotein B
28	APOC3	Apolipoprotein C-III
29	APOD	Apolipoprotein D
30	APOE	Apolipoprotein E
31	AQP1	Aquaporin 1 (Colton blood group)
32	ARF3	ADP-ribosylation factor 3
33	ARF4	ADP-ribosylation factor 4
34	ARF6	ADP-ribosylation factor 6
35	ARL15	ADP-ribosylation factor-like 15
36	ARL8B	ADP-ribosylation factor-like 8B
37	ARPC2	Actin-related protein 2/3 complex, subunit 2, 34 kDa
38	ARPC3	Actin-related protein 2/3 complex, subunit 3, 21 kDa
39	ARPC4	Actin-related protein 2/3 complex, subunit 4, 20 kDa
40	ARPC5	Actin-related protein 2/3 complex, subunit 5, 16 kDa
41	BCAM	Basal cell adhesion molecule (Lutheran blood group)

42	BLMH	Bleomycin hydrolase
43	BLVRB	Biliverdin reductase B
44	BRK1	BRICK1, SCAR/WAVE actin nucleation complex subunit
45	BROX	Contains BRO1 domain and CAAX motif
46	C1QA	Complement component 1, q subcomponent, chain A
47	C1QB	Complement component 1, q subcomponent, chain B
48	C1QC	Complement component 1, q subcomponent, chain C
49	C1R	Complement component 1, r subcomponent
50	C1S	Complement component 1, s subcomponent
51	C4BPA	Complement component 4 binding protein, alpha
52	CAB39	Calcium-binding protein 39
53	CAMP	Antimicrobial peptide cathelicidin
54	CAND1	Cullin-associated and dissociated with neddylation 1
55	CAP1	CAP, adenylate cyclase-associated protein 1 (yeast)
56	CBR3	Carbonyl reductase 3
57	CCM2	Cerebral cavernous malformation 2
58	CD2AP	CD2-associated protein
59	CD36	CD36 molecule (thrombospondin receptor)
60	CD44	CD44 molecule (Indian blood group)
61	CD47	CD47 molecule
62	CD63	CD63 molecule
63	CD82	CD82 molecule
64	CD9	CD9 molecule
65	CDC37	Cell division cycle 37
66	CDC42	Cell division cycle 42
67	CDSN	Corneodesmosin
68	CETP	Cholesteryl ester transfer protein, plasma
69	CHMP5	Charged multivesicular body protein 5
70	CHMP6	Charged multivesicular body protein 6
71	CIB1	Calcium and integrin-binding protein 1 (calmyrin)
72	CLIC1	Chloride intracellular channel 1
73	CLIC4	Chloride intracellular channel 4
74	CMIP	C-Maf inducing protein
75	CNDP2	CNDP dipeptidase 2 (metallopeptidase M20 family)
76	COTL1	Coactosin-like F-actin binding protein 1
77	CPN2	Carboxypeptidase N, polypeptide 2
78	CPNE1	Copine I
79	CPNE3	Copine III
80	CRKL	v-crk sarcoma virus CT10 oncogene homolog
81	DAB2	Dab, mitogen-responsive phosphoprotein, homolog 2 (Drosophila)
82	DBNL	Drebrin-like protein
83	DCD	Dermcidin
84	DIP2B	Disco-interacting protein 2 homolog B (Drosophila)
85	DNM1L	Dynamin 1-like
86	DPP4	Dipeptidyl peptidase 4
87	DSC1	Desmocollin 1
88	DSC3	Desmocollin 3

89	DSG1	Desmoglein 1
90	EFNB1	Ephrin-B1
91	EFR3A	EFR3 homolog A (S. cerevisiae)
92	EGF	Epidermal growth factor
93	EHD1	EH domain-containing protein 1
94	EHD3	EH domain-containing protein 3
95	EHD4	EH domain-containing protein 4
96	EPS15	Epidermal growth factor receptor pathway substrate 15
97	EPS8	Epidermal growth factor receptor pathway substrate 8
98	ERP44	Endoplasmic reticulum protein 44
99	ESAM	Endothelial cell adhesion molecule
100	FCGBP	Fc fragment of IgG binding protein
101	FHL1	Four and a half LIM domains 1
102	FLNA	Filamin A, alpha
103	FLOT1	Flotillin 1
104	FLOT2	Flotillin 2
105	FN3K	Fructosamine-3-kinase
106	G6PD	Glucose-6-phosphate dehydrogenase
107	GANAB	Glucosidase, alpha; neutral AB
108	GGCT	Gamma-glutamylcyclotransferase
109	GGT1	Gamma-glutamyltransferase 1
110	GNA11	Guanine nucleotide-binding protein (G protein), alpha 11 (Gq class)
111	GNA13	Guanine nucleotide-binding protein (G protein), alpha 13
112	GNAI1	Guanine nucleotide-binding protein (G protein), inhibitory alpha 1 polypeptide
113	GNAI2	Guanine nucleotide-binding protein (G protein), inhibitory alpha 2 polypeptide
114	GNAI3	Guanine nucleotide-binding protein (G protein), inhibitory alpha 3 polypeptide
115	GNAQ	Guanine nucleotide-binding protein (G protein), q polypeptide
116	GNAZ	Guanine nucleotide-binding protein (G protein), alpha-z polypeptide
117	GP1BA	Glycoprotein Ib (platelet), alpha polypeptide
118	GPX1	Glutathione peroxidase 1
119	GRB2	Growth factor receptor-bound protein 2
120	GRHPR	Glyoxylate reductase/hydroxypyruvate reductase
121	GSTO1	Glutathione S-transferase omega 1
122	GSTP1	Glutathione S-transferase pi 1
123	HBB	Hemoglobin, beta
124	HBD	Hemoglobin, delta
125	HEG1	Heart development protein with EGF-like domains 1
126	HGS	Hepatocyte growth factor-regulated tyrosine kinase substrate
127	HRG	Histidine-rich glycoprotein
128	HSPB1	Heat shock protein 27kDa 1
129	ICAM2	Intercellular adhesion molecule 2
130	IDE	Insulin-degrading enzyme
131	IGHA1	Immunoglobulin heavy constant alpha 1
132	IGHA2	Immunoglobulin heavy constant alpha 2 (marker A2m)
133	IGHG1	Immunoglobulin heavy constant gamma 1 (marker G1m)

134	IGHG2	Immunoglobulin heavy constant gamma 2 (marker G2m)
135	IGHG3	Immunoglobulin heavy constant gamma 3 (marker G3m)
136	IGHG4	Immunoglobulin heavy constant gamma 4 (marker G4m)
137	IGHM	Immunoglobulin heavy constant mu
138	IGKC	Immunoglobulin kappa constant
139	IGLC2	Immunoglobulin lambda constant 2 (marker Kern-Oz)
140	IGLC6	Immunoglobulin lambda constant 6 (marker Kern+Oz-, gene/pseudogene)
141	IGLL5	Immunoglobulin lambda-like polypeptide 5
142	IGSF8	Immunoglobulin superfamily, member 8
143	ILK	Integrin-linked kinase
144	IMPA1	Inositol(myo)-1(or 4)-monophosphatase 1
145	INF2	Inverted formin, containing FH2 and WH2 domains
146	IST1	Increased sodium tolerance 1 homolog (yeast)
147	ITCH	Itchy E3 ubiquitin protein ligase
148	ITIH1	Inter-alpha-trypsin inhibitor heavy chain 1
149	ITIH2	Inter-alpha-trypsin inhibitor heavy chain 2
150	ITIH4	Inter-alpha-trypsin inhibitor heavy chain family member 4
151	KALRN	Kalirin, RhoGEF kinase
152	KNG1	Kininogen 1
153	LAMP1	Lysosomal-associated membrane protein 1
154	LASP1	LIM and SH3 protein 1
155	LBP	Lipopolysaccharide-binding protein
156	LCK	Proto-oncogene LCK, Src family tyrosine kinase
157	LDHA	Lactate dehydrogenase A
158	LDHB	Lactate dehydrogenase B
159	LIMS1	LIM domain and senescent cell antigen-like domains 1
160	LMAN1	Lectin, mannose-binding, 1
161	LMAN2	Lectin, mannose-binding 2
162	LRBA	Vesicle trafficking involved in LPS response, containing BEACH and anchor domains
163	LRP1	Low-density lipoprotein receptor-related protein 1
164	LSR	Lipolysis-stimulated lipoprotein receptor
165	LYN	Proto-oncogene LYN, Src family tyrosine kinase
166	MGLL	Monoacylglycerol lipase
167	MINK1	Misshapen-like kinase 1
168	MLEC	Malectin
169	MOB1B	MOB kinase activator 1B
170	MTPN	Myotrophin
171	MVP	Major vault protein
172	MYADM	Myeloid-associated differentiation marker
173	MYH14	Myosin, heavy chain 14, non-muscle
174	MYH9	Myosin, heavy chain 9, non-muscle
175	MYL6	Myosin, light chain 6, basic, smooth muscle, and non-muscle
176	MYL9	Myosin, light chain 9, regulatory
177	MYO1C	Myosin IC
178	NCK2	NCK adaptor protein 2
179	NSF	N-ethylmaleimide-sensitive factor

180	NUDT5	Nudix motif (nucleoside diphosphate-linked moiety X)-containing 5
181	OLA1	Obg-like ATPase 1
182	OSTF1	Osteoclast-stimulating factor 1
183	PARK7	Parkinson protein 7
184	PCBP1	Poly(rC)-binding protein 1
185	PCBP2	Poly(rC)-binding protein 2
186	PDCD6	Programmed cell death 6
187	PDIA3	Protein disulfide isomerase family A, member 3
188	PDIA6	Protein disulfide isomerase family A, member 6
189	PEBP1	Phosphatidylethanolamine-binding protein 1
190	PEF1	Penta-EF-hand domain-containing 1
191	PGAM1	Phosphoglycerate mutase 1 (brain)
192	PGK1	Phosphoglycerate kinase 1
193	PGM1	Phosphoglucomutase 1
194	PI4KA	Phosphatidylinositol 4-kinase, catalytic, alpha
195	PIGR	Polymeric immunoglobulin receptor
196	PLCB3	Phospholipase C, beta 3 (phosphatidylinositol-specific)
197	PLCG2	Phospholipase C, gamma 2 (phosphatidylinositol-specific)
198	PLTP	Phospholipid transfer protein
199	PMVK	Phosphomevalonate kinase
200	PODXL	Podocalyxin-like
201	PON1	Paraoxonase 1
202	PPIA	Peptidylprolyl isomerase A (Cyclophilin A)
203	PPIB	Peptidylprolyl isomerase B (Cyclophilin B)
204	PPM1A	Protein phosphatase, Mg2+/Mn2+ dependent 1A
205	PRDX1	Peroxiredoxin 1
206	PRDX2	Peroxiredoxin 2
207	PRDX5	Peroxiredoxin 5
208	PRDX6	Peroxiredoxin 6
209	PTPRA	Protein tyrosine phosphatase, receptor type A
210	PTPRJ	Protein tyrosine phosphatase, receptor type J
211	PYGB	Phosphorylase, glycogen; brain
212	PYGL	Phosphorylase, glycogen; liver
213	RAB10	RAB10, member of RAS oncogene family
214	RAB13	RAB13, member of RAS oncogene family
215	RAB14	RAB14, member of RAS oncogene family
216	RAB18	RAB18, member of RAS oncogene family
217	RAB1A	RAB1A, member of RAS oncogene family
218	RAB1B	RAB1B, member of RAS oncogene family
219	RAB21	RAB21, member of RAS oncogene family
220	RAB2A	RAB2A, member of RAS oncogene family
221	RAB35	RAB35, member of RAS oncogene family
222	RAB4A	RAB4A, member of RAS oncogene family
223	RAB5A	RAB5A, member of RAS oncogene family
224	RAB5B	RAB5B, member of RAS oncogene family
225	RAB5C	RAB5C, member of RAS oncogene family
226	RAB6A	RAB6A, member of RAS oncogene family
227	RAB7A	RAB7A, member of RAS oncogene family

228	RAB8A	RAB8A, member of RAS oncogene family
229	RAB8B	RAB8B, member of RAS oncogene family
230	RAC1	Ras-related C3 botulinum toxin substrate 1 (Rho family, small GTP-binding protein Rac1)
231	RAC2	Ras-related C3 botulinum toxin substrate 2 (Rho family, small GTP-binding protein Rac2)
232	RAC3	Ras-related C3 botulinum toxin substrate 3 (Rho family, small GTP-binding protein Rac3)
233	RALA	v-ral simian leukemia viral oncogene homolog A (ras-related)
234	RALB	v-ral simian leukemia viral oncogene homolog B
235	RAN	RAN, member of RAS oncogene family
236	RAP1A	RAP1A, member of RAS oncogene family
237	RAP1B	RAP1B, member of RAS oncogene family
238	RAP2A	RAP2A, member of RAS oncogene family
239	RAP2B	RAP2B, member of RAS oncogene family
240	RENBP	Renin-binding protein
241	RHEB	Ras homolog enriched in brain
242	RHOA	Rho family homolog A
243	RHOC	Rho family homolog C
244	RHOF	Rho family homolog F (in filopodia)
245	RHOG	Rho family homolog G
246	RNF11	Ring finger protein 11
247	RPN2	Ribophorin II
248	RRAS	Related RAS viral oncogene homolog (r-ras)
249	RRAS2	Related RAS viral oncogene homolog 2
250	SND1	Staphylococcal nuclease and Tudor domain-containing 1
251	SNX18	Sorting nexin 18
252	SNX3	Sorting nexin 3
253	SRC	Proto-oncogene SRC, non-receptor tyrosine kinase
254	STAM2	Signal transducing adapter molecule (SH3 domain and ITAM motif) 2
255	STAT3	Signal transducer and activator of transcription 3 (acute-phase response factor)
256	STK10	Serine/threonine kinase 10
257	STK24	Serine/threonine kinase 24
258	STK38	Serine/threonine kinase 38
259	STOM	Stomatin
260	STRAP	Serine/threonine kinase receptor-associated protein
261	STX11	Syntaxin 11
262	STX12	Syntaxin 12
263	STX4	Syntaxin 4
264	STX7	Syntaxin 7
265	SYTL4	Synaptotagmin-like protein 4
266	TAOK1	TAO kinase 1
267	TAOK3	TAO kinase 3
268	TF	Transferrin
269	TFG	TRK-fused gene
270	TGM3	Transglutaminase 3

271	TKT	Transketolase
272	TLN1	Talin 1
273	TMED9	Transmembrane emp24 domain-containing protein 9
274	TNPO1	Transportin 1
275	TPM4	Tropomyosin 4
276	TPP2	Tripeptidyl peptidase II
277	TPST2	Tyrosylprotein sulfotransferase 2
278	TTYH3	Tweety family member 3
279	TWF2	Twinfilin-2, actin-binding protein
280	TXNL1	Thioredoxin-like 1
281	UBA1	Ubiquitin-like modifier activating enzyme 1
282	UBE2N	Ubiquitin-conjugating enzyme E2N
283	USO1	Vesicle transport factor USO1
284	USP9X	Ubiquitin-specific peptidase 9, X-linked
285	VAMP2	Vesicle-associated membrane protein 2 (synaptobrevin 2)
286	VAMP3	Vesicle-associated membrane protein 3
287	VAMP7	Vesicle-associated membrane protein 7
288	VAMP8	Vesicle-associated membrane protein 8
289	VAT1	Vesicular amine transporter 1
290	VDAC2	Voltage-dependent anion channel 2
291	VNN1	Vanin 1
292	VPS25	Vacuolar protein sorting-associated protein 25 (S. cerevisiae homolog)
293	VPS28	Vacuolar protein sorting-associated protein 28 (S. cerevisiae homolog)
294	VPS36	Vacuolar protein sorting-associated protein 36 (S. cerevisiae homolog)
295	VPS4A	Vacuolar protein sorting-associated protein 4 homolog A (S. cerevisiae)
296	VPS4B	Vacuolar protein sorting-associated protein 4 homolog B (S. cerevisiae)
297	VTA1	Vesicle trafficking protein VTA1
298	VTI1B	Vesicle transport through interaction with t-SNAREs homolog 1B
299	VWF	Von Willebrand factor
300	WASF2	WASP family member 2
301	WBP2	WW domain-binding protein 2
302	WDR1	WD repeat domain 1
303	WWP1	WW domain-containing E3 ubiquitin-protein ligase 1
304	YKT6	YKT6 v-SNARE homolog (S. cerevisiae)
