# Supplementary material for: The Proteomic Analysis of Platelet Extracellular Vesicles in Diabetic Patients by nanoLC-MALDI-MS/MS and nanoLC-TIMS-MS/MS
Source: Molecules. 2025 Mar 20;30(6):1384. doi: 10.3390/molecules30061384 (PMC11944696; doi:10.3390/molecules30061384)
Supplement: Supplementary file 1 [file molecules-30-01384-s001.zip › Supplementary files/Table S3.pdf]

Table S3: List of analyzed PEV proteins with differential expression in women with diabetes (Figure 6).

Gene	Protein Name	Disease Related (MIM)	Change	log2 Fold Change	p-value
IGLC2	Immunoglobulin lambda constant 2	-	↑	2.289	1.1239
CDH1	Cadherin-1	Blepharo-cheilo-dontic syndrome 1 (119580); Breast cancer, lobular, somatic 1 (14480); Diffuse gastric and lobular breast cancer syndrome (137215); Endometrial cancer (608089); Ovarian cancer (167000); Prostate cancer susceptibility (176807)	↑	2.26	1.3866
CFI	Complement factor I	Complement factor I deficiency (610984); Atypical hemolytic-uremic syndrome (612923); Age-related macular degeneration susceptibility (615439)	↑	2.259	1.2412
AHCY	Adenosylhomocysteinase	Hypermethioninemia with S-adenosylhomocysteine hydrolase deficiency (613752)	↑	2.2303	1.2736
GAS6	Growth arrest-specific protein 6	-	↑	1.9256	1.962
PTER	Phosphotriesterase-related protein	-	↑	1.8453	1.3096
BDH2	3-hydroxybutyrate dehydrogenase type 2	-	↑	1.7911	1.1196
ISLR	Immunoglobulin superfamily containing leucine-rich repeat protein	-	↑	1.73	1.3282
GRHPR	Glyoxylate reductase/hydroxypyruvate reductase	Primary hyperoxaluria type II (260000)	↑	1.6992	1.0588
KLK1	Kallikrein-1	Kallikrein urinary activity deficiency (615953)	↑	1.6508	1.3752
MMRN2	Multimerin-2	-	↑	1.5796	1.5547
CILP2	Cartilage intermediate layer protein 2	-	↑	1.5721	1.951
CLEC3B	Tetranectin	Macular dystrophy (619977)	↑	1.5719	1.8386
ST3GAL6	Type 2 lactosamine alpha-2,3-sialyltransferase	-	↑	1.5163	1.0545
PVR	Poliovirus receptor	-	↑	1.423	2.7101
CNTN1	Contactin-1	Congenital myopathy 12 (612540)	↑	1.3721	1.8254
AKR7A2	Aflatoxin B1 aldehyde reductase member 2	-	↑	1.3139	1.0286
ZG16B	Zymogen granule protein 16 homolog B	-	↑	1.2464	1.0456

B3GNT2	N-acetyllactosaminide beta-1,3-N-acetylglucosaminyltransferase 2	-	↑	1.2343	1.4653
LTBP2	Latent-transforming growth factor beta-binding protein 2	Weill-Marchesani syndrome 3 (614819); Primary congenital glaucoma 3D (613086); Microspherophakia with ectopia lentis and secondary glaucoma (251750)	↑	1.2256	1.1075
SERPINF2	Alpha-2-antiplasmin	Alpha-2-plasmin inhibitor deficiency (262850)	↑	1.157	1.4657
PPIC	Peptidyl-prolyl cis-trans isomerase C	-	↑	1.0581	1.0801
TTC38	Tetratricopeptide repeat protein 38	-	↑	1.0467	1.8947
ATP6AP1	V-type proton ATPase subunit S1	Immunodeficiency 47 (300972)	↑	1.023	1.591
EEF1G	Elongation factor 1-gamma	-	↑	0.9706	1.8069
IL6ST	Interleukin-6 receptor subunit beta	Immunodeficiency 94 with autoinflammation and dysmorphic facies (619750); Recurrent hyper-IgE infections syndrome 4A, autosomal dominant (619752); Recurrent hyper-IgE infections syndrome 4B, autosomal recessive (618523); Stuve-Wiedemann syndrome 2 (619751)	↑	0.9603	1.0062
ARHGEF10L	Rho guanine nucleotide exchange factor 10-like protein	-	↑	0.9396	2.0202
CCT7	T-complex protein 1 subunit eta	-	↑	0.9133	1.6054
LAP3	Cytosol aminopeptidase	-	↑	0.899	1.0229
UGP2	UTP--glucose-1-phosphate uridylyltransferase	Developmental and epileptic encephalopathy 83 (618744)	↑	0.8975	1.3654
PSMD11	26S proteasome non-ATPase regulatory subunit 11	-	↑	0.8933	1.0876
ITFG1	T-cell immunomodulatory protein	-	↑	0.8909	1.4099
OPLAH	5-oxoprolinase	5-oxoprolinase deficiency (260005)	↑	0.8768	1.2917
CCT6A	T-complex protein 1 subunit zeta	-	↑	0.8677	1.133
EPHB6	Ephrin type-B receptor 6	-	↑	0.857	1.7903
GK	Glycerol kinase	Glycerol kinase deficiency (307030)	↑	0.8159	1.0094
SDC4	Syndecan-4	-	↑	0.8133	1.7987
FN1	Fibronectin	Glomerulopathy with fibronectin deposits 2 (601894); Spondylometaphyseal dysplasia corner fracture type (184255)	↑	0.7579	1.5832
PTPRS	Receptor-type tyrosine-protein phosphatase S	-	↑	0.7432	1.0614

PCDHGC3	Protocadherin gamma-C3	-	↑	0.6953	1.274
VWF	von Willebrand factor	von Willebrand disease, type 1 (193400); von Willebrand disease, type 3 (277480); von Willebrand disease, types 2A, 2B, 2M, and 2N (613554)	↑	0.6704	1.2869
PPP2R2A	Serine/threonine-protein phosphatase 2A 55 kDa regulatory subunit B alpha isoform	-	↑	0.6544	1.0663
CCT8	T-complex protein 1 subunit theta	-	↑	0.6309	1.1888
MAT2A	S-adenosylmethionine synthase isoform type-2	-	↑	0.6044	1.2059
AFMID	Kynurenine formamidase	-	↑	0.5407	1.5052
SPAG9	C-Jun-amino-terminal kinase-interacting protein 4	-	↑	0.51	1.0788
FCGBP	IgGFc-binding protein	-	↑	0.4784	1.0034
RPS8	40S ribosomal protein S8	-	↑	0.4342	2.8191
HAGH	Hydroxyacylglutathione hydrolase, mitochondrial	Glyoxalase II deficiency (614033)	↑	0.402	1.4726
PAICS	Bifunctional phosphoribosylaminoimidazole carboxylase/phosphoribosylaminoimidazole succinocarboxamide synthetase	Phosphoribosylaminoimidazole carboxylase deficiency (619859)	↑	0.3516	1.0159
NAPRT	Nicotinate phosphoribosyltransferase	-	↑	0.3225	1.2903
FKBP4	Peptidyl-prolyl cis-trans isomerase FKBP4	-	↑	0.2971	2.3123
SELENBP1	Methanethiol oxidase	Extraoral halitosis due to MTO deficiency (618148)	↓	-0.4081	1.1085
NME1-NME2	Nucleoside diphosphate kinase	-	↓	-0.4259	2.1903
SCRN1	Secernin-1	-	↓	-0.4661	1.1105
USP5	Ubiquitin carboxyl-terminal hydrolase 5	-	↓	-0.4866	1.1734
AP1B1	AP-1 complex subunit beta-1	Keratitis-ichthyosis-deafness syndrome, autosomal recessive (242150)	↓	-0.4986	1.508
PMVK	Phosphomevalonate kinase	Porokeratosis 1, multiple types (175800)	↓	-0.529	1.0031
PACSIN2	Protein kinase C and casein kinase substrate in neurons protein 2	-	↓	-0.5926	1.181
TXNDC17	Thioredoxin domain-containing protein 17	-	↓	-0.6774	2.3686
PLS1	Plastin-1	Deafness, autosomal dominant 76 (618787)	↓	-0.6834	1.5477

COMT	Catechol O-methyltransferase	Panic disorder susceptibility (167870); Schizophrenia susceptibility (181500)	↓	-0.7746	1.7662
MVP	Major vault protein	-	↓	-0,7832	1,015
GLO1	Lactoylglutathione lyase	-	↓	-0,8566	1,4808
CMPK1	UMP-CMP kinase	-	↓	-0,9797	1,1151
HEBP1	Heme-binding protein 1	-	↓	-0,9877	1,1434
MTPN	Myotrophin	-	↓	-0,9889	1,1322
PRKAR2A	cAMP-dependent protein kinase type II-alpha regulatory subunit	-	↓	-0,9894	1,1053
PAFAH1B2	Platelet-activating factor acetylhydrolase IB subunit alpha2	-	↓	-0,9941	1,1767
CTDSP1	Carboxy-terminal domain RNA polymerase II polypeptide A small phosphatase 1	-	↓	-1,0345	1,0796
GDPD3	Lysophospholipase D GDPD3	-	↓	-1,0899	1,0002
DENND2D	DENN domain-containing protein 2D	-	↓	-1,1147	1,3213
SCIN	Scinderin	-	↓	-1,1271	1,7852
AK1	Adenylate kinase isoenzyme 1	Hemolytic anemia due to adenylate kinase deficiency (612631)	↓	-1.2108	1.5576
PRKCD	Protein kinase C delta type	Autoimmune lymphoproliferative syndrome type III (615559)	↓	-1.2552	1.1124
RRAS	Ras-related protein R-Ras	-	↓	-1.3344	1.0763
TMSB4X	Thymosin beta-4	-	↓	-1.491	1.0502
PATJ	InaD-like protein	-	↓	-1.504	1.1997
ITLN1	Intelectin-1	-	↓	-1.5095	1.0146
C4A	Complement C4-A	C4a deficiency (614380); Blood group, Rodgers (614374)	↓	-1.5172	1.1069
FLOT2	Flotillin-2	-	↓	-1.5794	1.1733
FLOT1	Flotillin-1	-	↓	-1.6028	1.3419
C16orf89	UPF0764 protein C16orf89	-	↓	-1.6799	1.2079
