# Supplementary material for: The Proteomic Analysis of Platelet Extracellular Vesicles in Diabetic Patients by nanoLC-MALDI-MS/MS and nanoLC-TIMS-MS/MS
Source: Molecules. 2025 Mar 20;30(6):1384. doi: 10.3390/molecules30061384 (PMC11944696; doi:10.3390/molecules30061384)
Supplement: Supplementary file 1 [file molecules-30-01384-s001.zip › Supplementary files/Table S4.pdf]

Table S4: List of analyzed PEV proteins with differential expression in men with diabetes (Figure 7).

Gene	Protein Name	Disease Related (MIM)	Change	log2 Fold Change	p-value
UMOD	Uromodulin	Autosomal dominant tubulointerstitial kidney disease 1 (162000)	↑	3.1899	1.2458
RIDA	2-iminobutanoate/2-iminopropanoate deaminase	-	↑	1.702	1.2176
TGM2	Protein-glutamine gamma-glutamyltransferase 2	-	↑	1.5677	1.4664
TRPV5	Transient receptor potential cation channel subfamily V member 5	-	↑	1.4339	1.0187
GLRX	Glutaredoxin-1	-	↑	1.3912	1.4329
PDE8A	High affinity cAMP-specific and IBMX-insensitive 3',5'-cyclic phosphodiesterase 8A	-	↑	1.0027	1.2929
STAT3	Signal transducer and activator of transcription 3	Multisystem autoimmune disease, infantile-onset, 1 (615952); Hyper-IgE recurrent infection syndrome (147060)	↑	0.6805	1.3426
NAMPT	Nicotinamide phosphoribosyltransferase	-	↑	0.4596	1.0472
TPPP3	Tubulin Polymerization-Promoting Protein Family Member 3	-	↑	0.443	1.0154
EVPL	Envoplakin	-	↓	-0.5218	1.1352
ATP12A	Potassium-transporting ATPase alpha-2 chain	-	↓	-0.5304	1.0486
PACSIN2	Protein kinase C and casein kinase substrate in neurons protein 2	-	↓	-0.7019	1.0553
CTSH	Pro-cathepsin H	-	↓	-0.7258	1.0047
PLS1	Plastin-1	Deafness, autosomal dominant 76 (618787)	↓	-0.824	1.0216
ALDH1A1	Aldehyde dehydrogenase 1A1	-	↓	-0.832	1.1657
CDHR5	Cadherin-related family member 5	-	↓	-0.8475	1.8142
SLC22A12	Solute carrier family 22 member 12	Renal hypouricemia (220150)	↓	-0.9646	1.2315
CLIC6	Chloride intracellular channel protein 6	-	↓	-0.9783	1.0518

SLC47A1	Multidrug and toxin extrusion protein 1	-	↓	-0.9989	1.0588
SEPTIN2	Septin-2	-	↓	-1.0301	1.1147
CRIP2	Cysteine-rich protein 2	-	↓	-1.0851	1.0393
TTC38	Tetratricopeptide repeat protein 38	-	↓	-1.0976	1.4747
MGAM	Maltase-glucoamylase	-	↓	-1.1408	1.5966
KHK	Ketohexokinase	Essential fructosuria (229800)	↓	-1.216	1.0351
DAB2	Disabled homolog 2	-	↓	-1.2353	2.1022
LAMB1	Laminin subunit beta-1	Lissencephaly 5 (615191)	↓	-1.2356	1.142
RNASE2	Non-secretory ribonuclease	-	↓	-1.2379	1.7826
TWF1	Twinfilin-1	-	↓	-1.2732	1.0986
CLIC3	Chloride intracellular channel protein 3	-	↓	-1.2969	1.2753
UPK1B	Uroplakin-1b	-	↓	-1.2979	1.324
PCK1	Phosphoenolpyruvate carboxykinase, cytosolic [GTP]	Phosphoenolpyruvate carboxykinase deficiency, cytosolic (261680)	↓	-1.3273	1.2058
DBNL	Drebrin-like protein	-	↓	-1.3284	1.5219
TKFC	Triose kinase/FMN cyclase	Triose kinase and FMN cyclase deficiency syndrome (618805)	↓	-1.3297	1.1079
CTSD	Cathepsin D	Neuronal ceroid lipofuscinosis, 10 (610127)	↓	-1.3762	1.3757
LBP	Lipopolysaccharide-binding protein	-	↓	-1.3836	1.1899
AKR7A3	Aflatoxin B1 aldehyde reductase member 3	-	↓	-1.4582	1.143
CTTN	Cortactin	-	↓	-1.4648	1.3768
GPD1	Glycerol-3-phosphate dehydrogenase [NAD(+)], cytoplasmic	Transient infantile hypertriglyceridemia (614480)	↓	-1.515	1.1776
ACE2	Angiotensin-converting enzyme 2	-	↓	-1.5289	1.5088
CDH1	Cadherin-1	Hereditary diffuse gastric and lobular breast cancer syndrome (137215)	↓	-1.5775	1.4656
SUSD2	Sushi domain-containing protein 2	-	↓	-1.7052	1.1237
RBP4	Retinol-binding protein 4	Microphthalmia, isolated, with coloboma 10 (616428)	↓	-1.8336	1.0775
GPC3	Glypican-3	Simpson-Golabi-Behmel syndrome, type 1 (312870)	↓	-1.8716	1.2689
PIP	Prolactin-induced protein	-	↓	-1.9603	1.3399
C9	Complement component C9	C9 deficiency (613825)	↓	-1.9793	1.0636

LRP2	Low-density lipoprotein receptor-related protein 2	Donnai-Barrow syndrome (222448)	↓	-2.0129	1.1131
HYAL1	Hyaluronidase-1	Mucopolysaccharidosis type IX (601492)	↓	-2.0132	1.1182
EFEMP1	Extracellular matrix protein 1 containing fibulin EGF	Doyne honeycomb retinal dystrophy (126600)	↓	-2.0181	1.3563
JCHAIN	Immunoglobulin J chain	-	↓	-2.0503	1.6719
IGHV3OR16-9	Immunoglobulin heavy variable 3/OR16-9 (non-functional) (fragment)	-	↓	-2.0766	1.3133
IGHA2	Immunoglobulin heavy constant alpha 2	-	↓	-2.1064	1.3499
CPN2	Carboxypeptidase N subunit 2	-	↓	-2.1067	1.7464
SOD3	Extracellular superoxide dismutase [Cu-Zn]	-	↓	-2.1091	1.3326
TPP1	Tripeptidyl-peptidase 1	Neuronal ceroid lipofuscinosis 2 (204500)	↓	-2.1654	1.2318
IGHA1	Immunoglobulin heavy constant alpha 1	-	↓	-2.2996	1.9599
PIGR	Polymeric immunoglobulin receptor	-	↓	-2.4044	2.0525
CPVL	Probable carboxypeptidase CPVL	-	↓	-2.4151	1.4422
IGHV3OR16-12	Immunoglobulin heavy variable 3/OR16-12 (non-functional) (Fragment)	-	↓	-2.449	2.022
S100A8	Protein S100-A8	-	↓	-2.4581	1.2273
PGLYRP1	Peptidoglycan recognition protein 1	-	↓	-2.4774	1.094
HSPG2	Basement membrane-specific heparan sulfate proteoglycan core protein	Schwartz-Jampel syndrome, type 1 (255800)	↓	-2.4781	1.1545
IGHM	Immunoglobulin heavy constant mu	-	↓	-2.5154	1.7199
MMRN2	Multimerin-2	-	↓	-2.5176	1.9842
IGHV3-7	Immunoglobulin heavy variable 3-7	-	↓	-2.5353	1.3085
COL15A1	Collagen alpha-1(XV) chain	-	↓	-2.5612	1.1172
GSN	Gelsolin	Finnish-type amyloidosis (105120)	↓	-2.636	1.0696
C3	Complement C3	Atypical hemolytic-uremic syndrome (612925)	↓	-2.6478	2.1537
NEU1	Sialidase-1	Sialidosis type I (256550)	↓	-2.6538	1.309
Gene	Protein Name	Disease Related (MIM)	Change	log2 Fold Change	p-value
MAN1A1	Mannosyl-oligosaccharide 1,2-alpha-mannosidase IA	-	↓	-2.6855	1.1581

COL6A1	Collagen alpha-1(VI) chain	Bethlem myopathy 1 (158810), Ullrich congenital muscular dystrophy 1 (254090)	↓	-2.6893	1.1833
C4B_2	Complement C4-B	C4B deficiency (614379)	↓	-2.702	1.1407
ROBO4	Roundabout homolog 4	Aortic valve disease 3 (618496)	↓	-2.7483	1.0436
SERPINA1	Alpha-1-antitrypsin	-	↓	-2.8345	1.1394
SERPING1	Plasma protease C1 inhibitor	Hereditary angioedema types 1 and 2 (106100), Complement component 4 deficiency (120790)	↓	-2.9772	1.1755
KNG1	Kininogen-1	Hereditary angioedema (619363), High-molecular-weight kininogen deficiency (228960)	↓	-3.0032	1.3098
SERPINC1	Antithrombin-III	Thrombophilia 7 due to antithrombin III deficiency (613118)	↓	-3.0063	1.0199
CETP	Cholesteryl ester transfer protein	Hyperalphalipoproteinemia (143470)	↓	-3.2396	1.2805
ALB	Albumin	Hypertriiodothyroninemia dysalbuminemic (615999), Analbuminemia (616000)	↓	-3.2535	1.3672
PLG	Plasminogen	Hereditary angioedema (619360), Dysplasminogenemia (217090)	↓	-3.4185	1.3906
ITIH4	Inter-alpha-trypsin inhibitor heavy chain H4	-	↓	-3.4533	1.6204
NAGLU	Alpha-N-acetylglucosaminidase	Charcot-Marie-Tooth disease, axonal, type 2V (616491), Mucopolysaccharidosis type IIIB (Sanfilippo B) (252920)	↓	-3.6072	1.7457
MXRA8	Matrix remodeling-associated protein 8	-	↓	-3.674	1.0824
IGHG1	Immunoglobulin heavy constant gamma 1	-	↓	-3.9617	1.5675
IGLL5	Immunoglobulin lambda-like polypeptide 5	-	↓	-4.0277	3.5009
FGA	Fibrinogen alpha chain	Congenital afibrinogenemia (202400), Familial visceral amyloidosis (105200)	↓	-4.0611	1.1955
IGLC2	Immunoglobulin lambda constant 2	-	↓	-4.1931	2.6555
MASP2	Mannan-binding lectin serine protease 2	MASP2 deficiency (613791)	↓	-4.303	1.6394
AMBP	AMBP protein	-	↓	-4.3263	1.5097
IGHG2	Immunoglobulin heavy constant gamma 2	-	↓	-4.3524	2.3521
CD14	Monocyte differentiation antigen CD14	-	↓	-4.4269	1.6937
SERPINA3	Alpha-1-antichymotrypsin	Alpha-1-antichymotrypsin deficiency	↓	-4.464	1.4379
IGKV3-20	Immunoglobulin kappa variable 3-20	-	↓	-4.5118	2.5704

ANGPTL2	Angiopoietin-related protein 2	-	↓	-4.5599	1.1788
IGKC	Immunoglobulin kappa constant	Kappa light chain deficiency (614102)	↓	-4.6269	2.9701
LMAN2	Vesicular integral-membrane protein VIP36	-	↓	-5.1799	1.3136
